# Supplementary material for: Unveiling the role of local metabolic constraints on the structure and activity of spiking neural networks
Source: PLoS Comput Biol. 2025 Jun 13;21(6):e1013148. doi: 10.1371/journal.pcbi.1013148 (PMC12201681; doi:10.1371/journal.pcbi.1013148)
Supplement: S1 Text — (PDF) [file pcbi.1013148.s001.pdf]

Unveiling the role of local metabolic constraints on the structure and activity of spiking neural networks

Ismael Jaras[1,2*], Marcos E. Orchard[1], Pedro E. Maldonado[2,4], Rodrigo C. Vergara[3,4*]

1 Department of Electrical Engineering, Faculty of Mathematical and Physical Sciences, University of Chile, Santiago, Chile
2 Neurosystems Laboratory, Department of Neuroscience, Faculty of Medicine, University of Chile, Santiago, Chile
3 Departamento de Kinesiología, Facultad de Artes y Educación Física, Universidad Metropolitana de Ciencias de la Educación, Santiago, Chile.
4 Centro Nacional de Inteligencia Artificial CENIA, Santiago, Chile

* ismael.jaras@ing.uchile.cl * rodrigo.vergara_o@umce.cl

Supporting information

S1 fixed point stability analysis

Lets analyze the stability of the fixed point $(\langle \breve{w} \rangle, \langle \breve{A} \rangle, \langle \breve{\nu} \rangle)$ by introducing a small perturbation $(\delta w, \delta A, \delta \nu)$:

$$\begin{bmatrix} w \\ A \\ \nu \end{bmatrix} = \begin{bmatrix} \langle \breve{w} \rangle + \delta w \\ \langle \breve{A} \rangle + \delta A \\ \langle \breve{\nu} \rangle + \delta \nu, \end{bmatrix}$$

where $\langle x \rangle = N^{-1} \sum_k x_k$ denotes the population average of x.

The weight dynamics are governed by the average weight drift equation:

$$\overline{\Delta w_t} = \frac{1}{2\Delta t'} \int_{\Delta t'}^{\Delta t'} \Delta w dt = \frac{\lambda \tau_{syn}}{2\Delta t'} \left(1 - e^{-\frac{\Delta t'}{\tau_{syn}}}\right) \left[-\alpha + e^{-\eta \frac{A_H - A}{A_H}}\right],$$

and, for small $\Delta t'$ it can be approximated as:

$$\overline{\Delta w_t} \approx \frac{\lambda}{2} \left[-\alpha + e^{-\eta \frac{A_H - A}{A_H}}\right].$$

Thus, the weight dynamics around the fixed point is given by:

$$\begin{aligned} \overline{\Delta w_t}(w, A, \nu) &\approx \underbrace{\overline{\Delta w_t}(\langle \breve{w} \rangle, \langle \breve{A} \rangle, \langle \breve{\nu} \rangle)}_{=0} + \frac{\partial \overline{\Delta w_t}}{\partial w}\Big|_{x^*} \delta w + \frac{\partial \overline{\Delta w_t}}{\partial A}\Big|_{x^*} \delta A + \frac{\partial \overline{\Delta w_t}}{\partial \nu}\Big|_{x^*} \delta \nu, \\ &= \frac{\partial \overline{\Delta w_t}}{\partial A}\Big|_{x^*} \delta A, \\ &= \frac{\lambda \eta}{2 A_H} e^{-\eta \frac{A_H - \langle \breve{A} \rangle}{A_H}} \delta A, \end{aligned}$$

where $x^* = \langle \breve{w} \rangle, \langle \breve{A} \rangle, \langle \breve{\nu} \rangle$.

In a network with N neurons, the energy average level dynamic ($\frac{1}{T}\Delta\langle A\rangle = \overline{\Delta A}_t$) is governed by (see Eq. 41):

$$\overline{\Delta A}_t(\tilde{w}, A, \nu) = K(A_H - \langle A\rangle) - (m+1)E_{syn}N\langle\tilde{w}\rangle\langle\nu\rangle$$

Remembering that $\tilde{w} = |w/w_{max}|$, and analyzing the dynamics of A around the fixed point, we obtain:

$$\begin{aligned}
\overline{\Delta A}_t(\tilde{w}, A, \nu) \approx& \underbrace{\overline{\Delta A}_t(\breve{\tilde{w}}, \breve{A}, \breve{\nu})}_{=0} + \frac{\partial}{\partial w}\overline{\Delta A}_t\Big|_{x^*}\delta w \\
&+ \frac{\partial}{\partial A}\overline{\Delta A}_t\Big|_{x^*}\delta A + \frac{\partial}{\partial \nu}\overline{\Delta A}_t\Big|_{x^*}\delta\nu, \\
&= -\frac{(m+1)E_{syn}N\langle\breve{\nu}\rangle}{w_{max}}\delta w - K\delta A - \frac{(m+1)E_{syn}N\langle\breve{w}\rangle}{w_{max}}\delta\nu.
\end{aligned}$$

Finally, lets examine the firing rate $\langle\nu\rangle$ around the fixed point (see Eq. 25):

$$\begin{aligned}
\langle\breve{\nu}\rangle + \delta\nu \approx& \underbrace{\Phi(I_{stim} + N\langle\breve{w}\rangle\langle\breve{\nu}\rangle)}_{=\langle\breve{\nu}\rangle} + \frac{\partial}{\partial w}\Phi\Big|_{x^*}\delta w + \frac{\partial}{\partial \nu}\Phi\Big|_{x^*}\delta\nu, \\
\Rightarrow \delta\nu \approx& \frac{\partial}{\partial w}\Phi\Big|_{x^*}\delta w + \frac{\partial}{\partial \nu}\Phi\Big|_{x^*}\delta\nu, \\
=& \left(1 - \frac{\partial}{\partial\nu}\Phi\Big|_{x^*}\right)^{-1}\frac{\partial}{\partial w}\Phi\Big|_{x^*}\delta w.
\end{aligned}$$

Thus,

$$\frac{\delta\langle\nu\rangle}{T} = \overline{\Delta\nu}_t \approx \left(1 - \frac{\partial}{\partial\nu}\Phi\Big|_{x^*}\right)^{-1}\frac{\partial}{\partial w}\Phi\Big|_{x^*}\underbrace{\frac{\lambda\eta}{2A_H}e^{-\eta\frac{A_H - \langle\breve{A}\rangle}{A_H}}\delta A}_{\approx\overline{\Delta w}_t}.$$

Our analysis assumes that for small T, a change in a variable denoted x (Δx) can be approximated by the time derivative, *i.e.,* $\overline{\Delta x}_t \approx \dot{x}$. Using this approximation, we analyze the stability of the linearized system. The system dynamics around the fixed point can be express in matrix form as:

$$\begin{bmatrix}\overline{\Delta w}_t \\ \overline{\Delta A}_t \\ \overline{\Delta\nu}_t\end{bmatrix} = \begin{bmatrix} 0 & \frac{\lambda\eta}{2A_H}e^{-\eta\frac{A_H-\langle\breve{A}\rangle}{A_H}} & 0 \\ -\frac{(m+1)E_{syn}N\langle\breve{\nu}\rangle}{w_{max}} & -K & -\frac{(m+1)E_{syn}N\langle\breve{w}\rangle}{w_{max}} \\ 0 & \left(1-\frac{\partial\Phi}{\partial\nu}\right)^{-1}\frac{\partial\Phi}{\partial w}\frac{\lambda\eta}{2A_H}e^{-\eta\frac{A_H-\langle\breve{A}\rangle}{A_H}} & 0\end{bmatrix}\begin{bmatrix}\delta w \\ \delta A \\ \delta\nu\end{bmatrix}.$$

To determine whether the fixed point is stable, we need to examine the signs of the eigenvalues of M, which can be found by solving the characteristic equation $\det(M - \mu I) = 0$:

$$\begin{aligned}
0 =& -\mu\left[(\mu+K)\mu + \frac{(m+1)E_{syn}N\langle w\rangle}{w_{max}}\left(1 - \frac{\partial\Phi}{\partial\nu}\right)^{-1}\frac{\partial\Phi}{\partial w}\frac{\lambda\eta}{2A_H}e^{-\eta\frac{A_H-\langle A\rangle}{A_H}}\right] \\
&- \frac{\lambda\eta(m+1)E_{syn}N\langle\nu\rangle}{2A_H w_{max}}e^{-\eta\frac{A_H-\langle A\rangle}{A_H}}\mu, \\
0 =& -\mu^2(\mu+K) - \mu\underbrace{\frac{\lambda\eta(m+1)E_{syn}N}{2A_H w_{max}}e^{-\eta\frac{A_H-\langle A\rangle}{A_H}}}_{\xi}\left[\langle\nu\rangle + \langle w\rangle\underbrace{\left(1-\frac{\partial\Phi}{\partial\nu}\right)^{-1}\frac{\partial\Phi}{\partial w}}_{\varsigma}\right] \\
0 =& \mu\left(\mu^2 + \mu K + \xi(\varsigma\langle w\rangle + \langle\nu\rangle)\right) \qquad \text{(S1)}
\end{aligned}$$

There are three eigenvalues:

$$\mu_0 = 0,\ \mu_{1,2} = \frac{-K + \sqrt{K^2 - 4\xi(\varsigma\langle\breve{w}\rangle + \langle\breve{\nu}\rangle)}}{2} \tag{S2}$$

If the real part of μ_1 and μ_2 are negative, then $\mu_0 = 0$ implies that the systems is marginally stable around the fixed point. For the $\mu_{1,2}$ eigenvalues, since the term $4\xi(\sigma\langle\breve{w}\rangle + \langle\breve{\nu}\rangle)$ is positive (see S2 Fig), the real part of $\mu_{1,2}$ is negative, placing these roots in the negative half-plane.

To better understand how γ and η influence the system's behavior, we will study $\mu_{1,2}$ numerically as η and γ vary. For this numerical exploration, we make the following assumptions and simplifications:

1. We simplify the numerical calculations by approximating the neuron's activation function Φ_γ (see Fig 1) as:

$$\Phi_\varrho = \begin{cases} \nu_{max}\left[1 - e^{-x\varrho}\right] & \text{if } > 0 \\ 0 & \text{otherwise} \end{cases}$$

   In the simplified Φ_ϱ function, ϱ allows to modify the sensitivity of the neurons to different γ values. S1 Fig shows how Φ_ϱ behaves for different ϱ values. Thus, $\frac{\partial}{\partial x}\Phi(x) \approx \nu_{max}\varrho e^{-x\varrho}$. Allowing us to calculate ς.

2. we analyze the fixed point for $\langle\breve{\nu}\rangle > 0$. Given the form of Φ_ϱ, this requires $N\langle\breve{w}\rangle > 0$.

3. We calculate $\langle\breve{\nu}\rangle = \Phi_\varrho$. This requires estimating $N\langle\breve{w}\rangle\langle\breve{\nu}\rangle$. Using Eq. 41 (main text), we approximate:

$$\sum_k |w^k|\nu^k \approx \frac{-A_H K \ln(\alpha) w_{max}}{(m+1)\eta E_{syn}},$$
$$N\langle\breve{w}\rangle\langle\breve{\nu}\rangle \approx \frac{-A_H K \ln(\alpha) w_{max}}{(m+1)\eta E_{syn}}\pi,$$

   where $\pi < 1$. The π factor can be absorbed in ϱ when evaluating $\langle\nu\rangle = \Phi_\varrho(\sum_k w^k \nu^k)$.

S2 Fig shows the numerical evaluation of the average firing rate $\langle\breve{\nu}\rangle$ (first column), the term inside the square root in Eq. S2 $\Omega = K^2 - 4\xi(\varsigma\langle\breve{w}\rangle + \langle\breve{\nu}\rangle)$ (second column) and ς (third column) for a network of 500 neurons. Rows correspond to different values of K: $K = 1$ in first row, $K = 0.1$ in second row, and $K = 0.01$ in third row .

From panel **(h)**, we observe that for small K (severely impaired production), the networks may exhibit oscillatory behavior ($\Omega < 0$, complex conjugate roots). These oscillations can be mitigated by decreasing η or increasing γ. Additionally, examining panels **(b)**, **(e)**, and **(h)** reveal that the roots are more sensitive to changes in η compared to ϱ (analogous to γ), indicating the η variable has a greater influence on the system's behavior by altering the eigenvalues.

The third column (**(c)**, **(f)**, and **(i)**) shows that ς remains positive across all conditions. Therefore, the real part of $\mu_{1,2}$ eigenvalues is always negative.

List of Figures

S1 **Neurons' activation function $\Phi(x)$ approximation as** $\nu_{max}\left[1 - e^{-\varrho x}\right]$ . . . 5

S2 **Numerical evaluation of the system eigenvalues.** First row $K = 1$, second row $K = 0.5$, third row $K = 0.1$. $\Omega = K^2 - 4\xi(\varsigma\langle\breve{w}\rangle + \langle\breve{\nu}\rangle)$. . . . 6
