# Supplementary material for: Unveiling the role of local metabolic constraints on the structure and activity of spiking neural networks
Source: PLoS Comput Biol. 2025 Jun 13;21(6):e1013148. doi: 10.1371/journal.pcbi.1013148 (PMC12201681; doi:10.1371/journal.pcbi.1013148)
Supplement: S2 Text — (PDF) [file pcbi.1013148.s002.pdf]

# Unveiling the role of local metabolic constraints on the structure and activity of spiking neural networks

Ismael Jaras[1,2*], Marcos E. Orchard[1], Pedro E. Maldonado[2,4], Rodrigo C. Vergara[3,4*]

**1** Department of Electrical Engineering, Faculty of Mathematical and Physical Sciences, University of Chile, Santiago, Chile
**2** Neurosystems Laboratory, Department of Neuroscience, Faculty of Medicine, University of Chile, Santiago, Chile
**3** Departamento de Kinesiología, Facultad de Artes y Educación Física, Universidad Metropolitana de Ciencias de la Educación, Santiago, Chile.
**4** Centro Nacional de Inteligencia Artificial CENIA, Santiago, Chile

* ismael.jaras@ing.uchile.cl * rodrigo.vergara_o@umce.cl

## Supporting information

### S2 Average synaptic weight under different parameter conditions

To calculate the average synaptic weights shown in S3 Fig, we use the `weight recorder` provided by NEST (see `https://nest-simulator.readthedocs.io/en/v3.3/models/weight_recorder.html`). This device logs every weight change that occurs during each spike event. By collecting all the weight values recorded at each spike, we compute the average weight for that event, which reflects the overall evolution of synaptic strength over time.

# List of Figures

S3 **Average excitatory-excitatory synaptic weight values under different parameter conditions.** Each panel in the 3×3 grid shows a scatter of the average excitatory-excitatory synaptic weights for a specific combination of $(\gamma, \eta, K)$. **Top row:** $\gamma = 0$ and $K = 1$, with $\eta$ increasing from left to right (conditions shown in Fig 6). **Middle row:** $\eta = 50$ and $K = 1$, with $\gamma$ increasing from left to right (conditions shown in Fig 7). **Bottom row:** $\eta = 50$ and $\gamma = 20$, with $K$ decreasing from left to right (conditions shown in Fig 8). . . . . . . . . . . . . . . . . . . . . . . . . 3
