# Supplementary material for: Unveiling the role of local metabolic constraints on the structure and activity of spiking neural networks
Source: PLoS Comput Biol. 2025 Jun 13;21(6):e1013148. doi: 10.1371/journal.pcbi.1013148 (PMC12201681; doi:10.1371/journal.pcbi.1013148)
Supplement: S1 Table — (PDF) [file pcbi.1013148.s003.pdf]

S1 Table **Neuronal and synaptic parameters used when simulating the excitatory-inhibitory network.**

Parameter	Value	Unit
Δt_{sim}	0.1	ms
α	0.5	-
μ_{-}	0	-
μ_{+}	0	-
λ	0.01	-
τ_m	20	ms
τ_{ref}	8	ms
C_m	250	pF
v_{reset}	-70	mV
v_{rest}	-70	mV
v_{th}	-50	mV
τ_{syn}	6	ms
τ_{ap}	60	ms
τ_{syn}^{A}	60	ms
I_{stim}	166 ± 15	pA
E_{ap}	4.1	%
E_{syn}	0.5	%
E_{rp}	5	%
E_{hk}	5	%
