# Supplementary material for: Unveiling the role of local metabolic constraints on the structure and activity of spiking neural networks
Source: PLoS Comput Biol. 2025 Jun 13;21(6):e1013148. doi: 10.1371/journal.pcbi.1013148 (PMC12201681; doi:10.1371/journal.pcbi.1013148)
Supplement: S2 Table — (PDF) [file pcbi.1013148.s004.pdf]

S2 Table **Summary of K, γ and η parameter used in simulations.**

Figure n.	K	γ	η
Fig 5	1	0	0
Fig 6	1	0	$[30, 50, 100]$
Fig 7	1	$[10, 20, 100]$	50
Fig 8	$[0.7, 0.5, 0.1]$	20	50
