# Supplementary figures and images for: Unveiling the role of local metabolic constraints on the structure and activity of spiking neural networks
Source: PLoS Comput Biol. 2025 Jun 13;21(6):e1013148. doi: 10.1371/journal.pcbi.1013148 (PMC12201681; doi:10.1371/journal.pcbi.1013148)

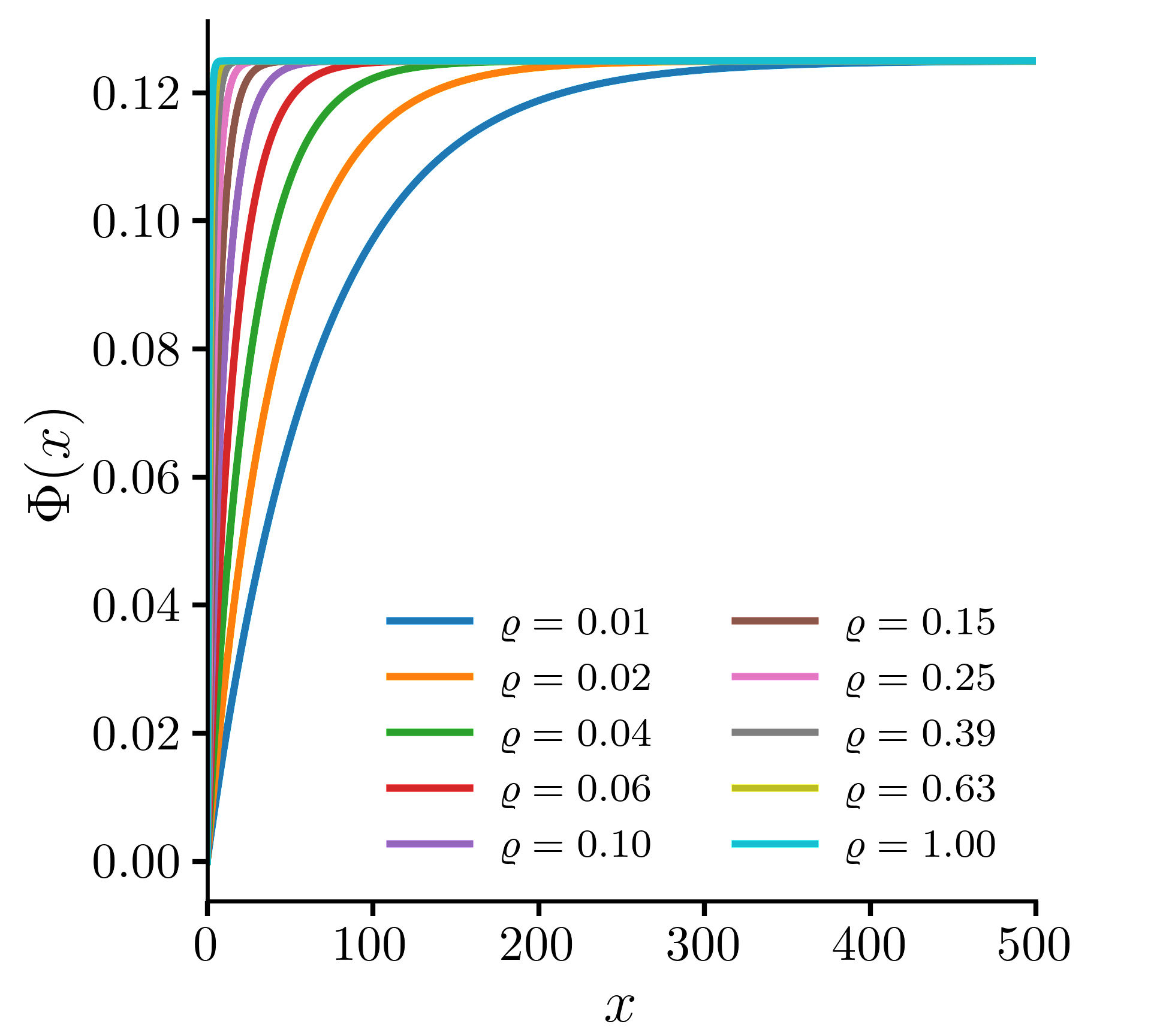

Supplement: S1 Fig — (TIF) [file pcbi.1013148.s005.tif]

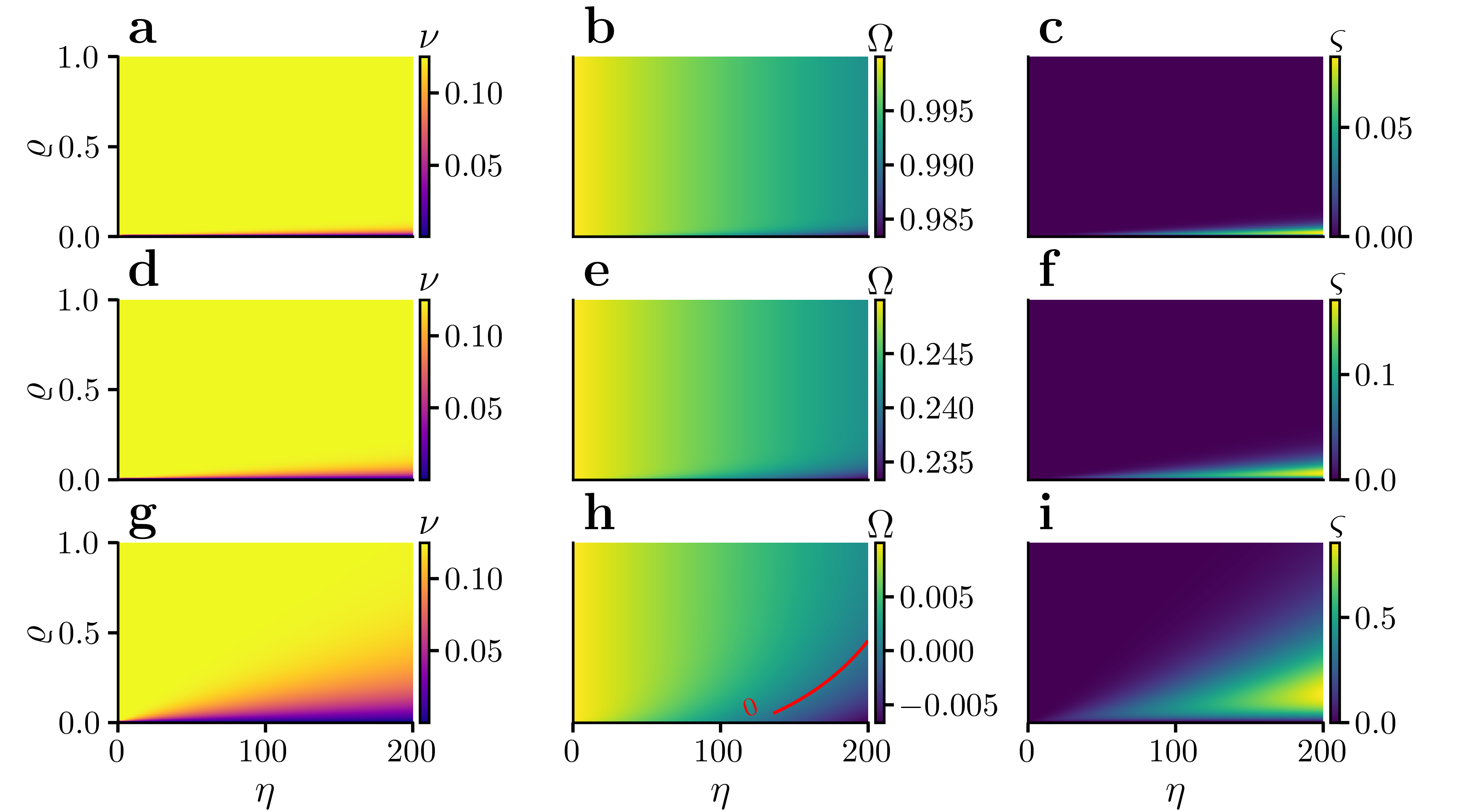

Supplement: S2 Fig — (TIF) [file pcbi.1013148.s006.tif]

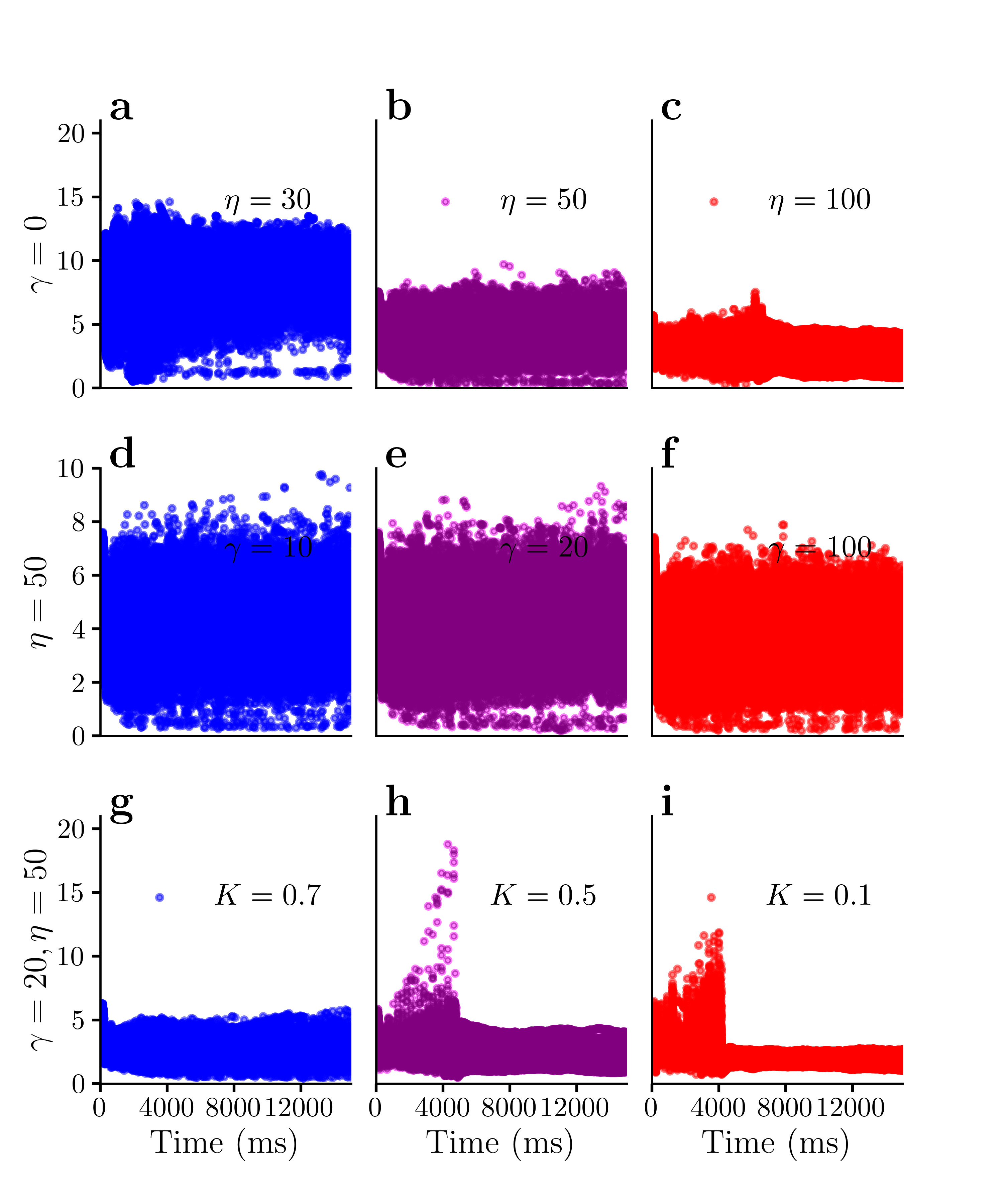

Supplement: S3 Fig — (TIF) [file pcbi.1013148.s007.tif]
