# Supplementary material for: Unveiling the role of local metabolic constraints on the structure and activity of spiking neural networks
Source: PLoS Comput Biol. 2025 Jun 13;21(6):e1013148. doi: 10.1371/journal.pcbi.1013148 (PMC12201681; doi:10.1371/journal.pcbi.1013148)
Supplement: Attachment — Submitted filename: review.pdf [file pcbi.1013148.s008.pdf]

Review PCOMPBIOL-D-23-01765

The model and its mathematical analysis seem novel and interesting. They would indeed contribute to advancing the understanding of how metabolic constraints contribute to shaping the activity of neuronal networks.

However, a detailed discussion of the reason behind the choices made in the models, their implications in terms of biological relevance is necessary, with at the very least some justification of the relevance of the model (= neuronal dynamics + EI balanced network) to specific cases of metabolic disruption. A justification of the values chosen for the parameters (notably E_{syn}), and their compatibility biological data on energy consumption in the brain (e.g. Howarth2012) must be provided. This may bring into question the hypothesis

$$E_{ap}\bar{\nu}_t \ll E_{syn}\sum_k \overline{w}_t^k \bar{\nu}_t^k$$

A discussion about how the models fit among the landscape of other neuronal and synaptic models including energetics would also be appreciated.

In addition, the Results section needs some major changes to be more synthetic and readable. Rather than selecting seemingly arbitrary values in Figures 8 to 16, the author should provide the whole phase diagram of the network model. The diagram should illustrate the main type of dynamics/attractors characterizing the network activity as a function of γ, η, and K. This would both provide a more principled analysis of the model and enable the reader graso most of the results in a single figure. The authors could then illustrate and discuss the different parts of the phase diagram using one figure for each type of activity (= region of phase space).

Scientific remarks

Regarding the neuronal model : I expect the main impact of ATP deficit, associated to the Na/K-pump, to involve the resting potential (i.e. via g_{leak}), rather than v_r. Though there can be changes in the reset potential during hypoxia, I am not sure: a) that this is a generic response that has been attributed directly to metabolic constraints, nor b) that it does not occur together with or later than the depolarization and with less influence on the neuronal activity (see e.g. Müller2000 and Chen2017). Of course, as the authors propose a phenomenological model, it can be fine to achieve a desired type of response to energy availability via a mechanism that differs from the biological one, but this should be explicitely mentioned and justified. This discussion was not present in the original article for the model; it would be good to have it now.

Similarly, the choice and relevance of an all-to-all network and an EI-balanced model should be discussed. Was this done purely out of convenience in terms of mathematical analysis or in terms of the type of brain regions and metabolic issues that the authors want to model? This is especially important as EI dynamics (notably asynchronous irregular, which is usually the one chosen for such networks) has very specific properties that have strong implications in terms of decorrelating the single cell properties and the network dynamics (see e.g. Fardet2020). On the other hands, multiple types of neurodegenerative diseases are known to be associated to rhythmic activities with properties that differ drastically from the EI model.

In the current state of the article, it is also unclear how important the energy in the neuronal model is (compared to the synaptic constraints) for shaping the network dynamics. This is especially problematic since the biologically-relevant range for the parameters is not discussed and the combination of values in Figure 12 do not seem to be the most relevant for that discussion.

Specific remarks

> Despite the significance of this relationship, specific details concerning the impact of metabolism on neuronal dynamics and neural network architecture remain elusive, creating a notable gap in the existing literature.

There are quite a few publications on this topic, particularly in the theoretical literature, that are not cited in the paper. This sentence, and the absence of mention of these other studies, may give a distorted view of the current state of the art.

> electric activity corresponds to 92% of neuron expenses [2]

I'm not sure what part of Jolivet et al. supports that claim. In addition, "electric activity" is unclear in that context and should be replaced with something clearer. As per Figure 2.C in [2], signaling-related energy usage is around 60% in neurons.

> Basically, realizing how crucial energy management is for shaping neural systems

That first part of the sentence should be removed.

> This study aims to advance our understanding of how local metabolic constraints influence the structure and activity of spiking neural networks. To achieve this, we introduce a novel energy-dependent plasticity rule and investigate the impact of local metabolic constraints through both analytical methods and numerical simulations.

This should either be moved up right after the sentences on EHP (and modified to fit within the 2nd-to-last paragraph) or removed altogether.

> Eq. 1

A_H and $A(t)$ should be defined (homeostatic and time-dependent ATP levels).

> Figure 1
>
> hy aren't the firing rates in the simulations following the precise values given by the mathematical formula? Is it due to the timestep of the simulation, the number of spikes considered or some other imprecision? This should be discussed or fixed. In particular, the blue and indigo curves should, in theory, not cross in the inset.
>
> Eq. 5

τ_{ref} should be defined in the text (refractory time); it might also be good to re-explicitate that the dependency in A comes from s_0 (thus making it obvious that τ_{ref} is a constant).

> Eq. 17

λ was already used as the firing rate in Eq. 5, please change one of the two for consistency.

> Eq. 18

α was already defined as $v_{rest}/v_{th} - 1$ in Eq. 2, please change one of the two for consistency.

> There is biological evidence suggesting the existence of excitatory-inhibitory (E-I) balanced networks in different parts of the brain, such as the CA3 region of the hippocampus, basal ganglia, and the primary visual cortex [27]

Citation [27] is a simulation study, please provide direct citations of biological papers asserting the relevance of balanced networks in each of these three regions (especially basal ganglia).

> Because both LTP and LTD depended on the activation of NMDA receptors and are absent in cases in which the postsynaptic neurons were GABAergic in nature [28], only excitatory-excitatory connections are plastic in our E-I network.

The plasticity of excitatory synapses on inhibitory postsynaptic neurons is a well-known phenomenon (Malenka2004, Lu2007). It has also been demonstrated that many types of synapses can display LTP, including inhibitory ones (Haas2006).

> To numerically test our theoretical predictions, the network is simulated utilizing the Neural Simulation Tool program (NEST) [29]

Please cite the specific version of NEST that was used: https://nest-simulator.readthedocs.io/en/stable/citing-nest.html The Scholarpedia article is not a relevant citation for

software.

> Figures 14C/15C

Do not use the average value in 15C but a 2D distribution. I would actually suggest using a distribution for Figures 8, 10, 13, and 15, both for consistency and because some other plots have values that are not completely stationnary.

> In addition, following in vitro measured weights strength in neuronal cell assemblies [32], initial synaptic strength values for the simulated network are drawn from an exponential distribution

Regarding network properties (Barral2016) or similar experimental works in vitro or in vivo would be more relevant given the specific model studied.

> Eq. 40

This does not seem correct, unless I misunderstandood how the matrices are constructed (in which case it should be explained in more details).

From what I understand, Eq. 39 is for a single neuron (let's call it (i$). It could (should ?) actually be written:

$$\overline{A}_t^i = A_H^i - \frac{1}{K}\left[E_{ap}\overline{\boldsymbol{\nu}}_t^i + E_{syn}\sum_j \overline{w}_t^{ij}\overline{\boldsymbol{\nu}}_t^j\right]$$

In that perspective, we can write the vector of activity on the set of all neurons $i \in [1, 500]$, i.e. $\overline{\mathbf{A}}_t = \{A_t^i\}_i$.

The only matrix involved is $\overline{\mathbf{w}} = \{\overline{w}_t^{ij}\}_{i,j}$ and the whole equation becomes:

$$\overline{\mathbf{A}}_t = \mathbf{A}_H - \frac{1}{K}[E_{ap}\overline{\boldsymbol{\nu}}_t + E_{syn}\overline{\mathbf{w}}_t\overline{\boldsymbol{\nu}}_t]$$

I.e.

$$\overline{\mathbf{A}}_t = \mathbf{A}_H - \frac{1}{K}[E_{ap}\mathbb{I} + E_{syn}\overline{\mathbf{w}}_t]\overline{\boldsymbol{\nu}}_t$$

Or, if one wants to transpose everything,

$$\overline{\mathbf{A}}_t^\top = \mathbf{A}_H^\top - \frac{1}{K}\overline{\boldsymbol{\nu}}_t^\top\left[E_{ap}\mathbb{I} + E_{syn}\overline{\mathbf{w}}_t^\top\right]$$

> it is possible to neglect the neuron’s own action potentials energy consumption

This is indeed the case with the values of E_{ap} and E_{syn}. However, it seems to directly contradict the results from [2] and (Howarth2012) where E_{ap}/E_{syn} is 20%/60%. As the values of E_{ap} and E_{syn} are currently not justified, it seems absolutely necessary to explain why these specific values where chosen.

> Exploring synaptic energy imbalances sensitivity

These paragraphs are really hard to read, even knowing what is going on. An additional schematics of the transition, rather than lengthy paragraphs, would probably make this much more understandable.

> Interestingly, when the synapses are highly sensitive to energy imbalance (Fig. 10E and 10F), it is not possible for all the neurons to achieve the metabolic fixed point, and some neurons achieve the silent fixed point previously described.

A more precise discussion of the mathematical transition for silenced neurons would be appreciated.

> Fig 9C (and more generally)

Mean and standard deviation are not relevant for a bimodal distribution, please plot each monomodal subpopulation separately.

Technical remarks

The provided code contains only the NESTML model (https://github.com/Wiss/nestml). It would be preferable to provide all the relevant code necessary to replicate the result in the study. Furthermore, rather than include the new model within a fork of the nestml repository (which requires to download 80MB of data to access 2 files of 3kB each), it would be better to include them in a separate repositories. I suggest uploading all the relevant code in the following form:

```
Jaras_et_al_MetabolicConstraintsModel/
├── README.md  # instructions to install the models and run the scripts
├── Models/
│   ├── ed_stdp_synapse.nestml
│   └── third_factor_stdp_synapse.nestml
└── Simulations/
    ├── fig1.py
    ├── ...
    └── fig16.py
```

This is just an example structure. Some figures may be grouped within a single file and some separate simulation files may be added to separate simulations and analysis whenever relevant.

Typographic remarks

Adapt the size of figures or the figures' fontsize such that the text in the figures appears similar to the main text.

There are issues with quotes. If LaTeX was used, make sure to use ``this'' and not "that" for quotes.

> where synaptic potentiation and depression balance each other in average.

on average
