# Supplementary material for: Unveiling the role of local metabolic constraints on the structure and activity of spiking neural networks
Source: PLoS Comput Biol. 2025 Jun 13;21(6):e1013148. doi: 10.1371/journal.pcbi.1013148 (PMC12201681; doi:10.1371/journal.pcbi.1013148)
Supplement: Attachment — Submitted filename: plos_answer_to_reviewers.pdf [file pcbi.1013148.s009.pdf]

**Reviewer #1:** NB: For a properly formatted version of this review, see the PDF attachment.

# Review PCOMPBIOL-D-23-01765

**Comment 1**
The model and its mathematical analysis seem novel and interesting. They would indeed contribute to advancing the understanding of how metabolic constraints contribute to shaping the activity of neuronal networks.

However, a detailed discussion of the reason behind the choices made in the models, their implications in terms of biological relevance is necessary, with at the very least some justification of the relevance of the model (= neuronal dynamics + EI balanced network) to specific cases of metabolic disruption.
A justification of the values chosen for the parameters (notably \(E_{syn}\)), and their compatibility biological data on energy consumption in the brain (e.g. [Howarth2012]) must be provided.
This may bring into question the hypothesis

Eapv¯t≪Esyn∑kw¯tkv¯tkE_{ap} \overline{\nu}_t \ll E_{syn} \sum_k \overline{w}_t^k \overline{\nu}^k_t

**Answer**
We thank the reviewer. In the following pages, we answer all specific reviewer's comments.

**Comment 2**
A discussion about how the models fit among the landscape of other neuronal and synaptic models including energetics would also be appreciated.

**Answer**
We chose the EDLIF model (Jaras et al., 2021) because, in our opinion, it is the simplest model that allows us to include energy dependencies. This simplicity allows a deep analytical exploration of the model's dynamics for the single-neuron case or when used to build neuronal networks. Despite its simplicity, the model can replicate interesting behaviors found in neurodegenerative diseases, such as the dead-loop observed in ALS (Le Masson et al., 2014). That being said, we acknowledge that other models can also be used to include energy dependencies (eLIF and mAdEx, Fardet et al., 2020). EDLIF, eLIF and mAdEx share the same fundamental rationale in the sense that a decrease in the available energy inhibits the Na/K pump function, thus leading to a sodium accumulation inside the cell. However, this phenomenon is differently represented in EDLIF, compared to eLIF and mAdEx. Particularly, in EDLIF the membrane voltage includes a simpler dependence on available energy, where the reset potential changes as a function of the available energy (partial repolarization mechanisms: $v_{reset} = \beta(A) v_{th}$, with an energy dependent reset parameter $\beta(A(t))$).

One drawback related to our model choice is the lack of a richer behavior repertoire at the neuron level. Nevertheless, part of this drawback could be addressed by a simple modification to EDLIF. In particular, by adding a minimal available energy requirement to

generate spikes, as present in eLIF and mAdEx, EDLIF could also show “depolarization block” behavior. Despite its simplicity, the EDLIF model has an interesting feature that is not present in eLIF and mAdEx models. In addition to the “energetic health” parameter ($K$ in EDLIF, and \alpha in eLIF and mAdEx), EDLIF includes a parameter controlling the sensitivity of the neuron to an energy imbalance ($\gamma$ in Eqn. 2). This parameter allows us to understand the impact of an energy imbalance considering different neurons’ sensitivity, thus it also allows us to differentiate how the same metabolic neuronal impairment affects two different neurons (it could be argued that a similar distinction could be made by modifying the $E_u$ or $E_f$ parameter in eLIF, although the interpretation of this modification is less direct).

Another distinction between EDLIF and eLIF (and mAdEx) models, is how energy consumption is modeled. Under the eLIF and mAdEx model, a spike produces an instantaneous decrease in the available energy, thus the dynamic of spikes’ energy consumption is reduced to an instant. On the other hand, under the EDLIF model, energy consumption has its own dynamic (Eqns. 10 and 12). This is an important distinction because energy consumption is not an instantaneous process and, in general, the time constants associated with neuronal energy consumption are much larger than the membrane time constants, and the rate at which energy is consumed impacts neurons’ behavior. This is particularly important when studying neuronal metabolic production impairment, as the same total energy consumed per activity could lead to different behaviors in the neuron if the rate at which that energy is consumed differs. Therefore, in our opinion, including energy consumption dynamics is closer to biology and is an important ingredient to study how energy dynamics affect neuronal behavior. Of course, those energy consumption dynamics could also be included in the eLIF and mAdEx models, and given their more rich repertoire of behavior, there is an opportunity to understand in more detail the impact of energy dynamics on neuronal behavior under those models. It would also be interesting to explore network dynamics using eLIF and mAdEx jointly with our ED-STDP rule.

The discussion has been updated including references for the eLIF and mAdEx models, as well as an explanation of our decision for using the EDLIF model.

**Comment 3**
In addition, the Results section needs some major changes to be more **synthetic and readable**. Rather than selecting seemingly arbitrary values in Figures 8 to 16, **the author should provide the whole phase diagram of the network model**.
The diagram should illustrate the main type of dynamics/attractors characterizing the network activity as a function of \(\gamma\), \(\eta\), and K.
This would both provide a more principled analysis of the model and enable the reader graso most of the results in a single figure.
The authors could then illustrate and discuss the different parts of the phase diagram using one figure for each type of activity (= region of phase space).

**Answer**
In Fig. 7 (new Fig. 4) the attractors characterizing the network activity as a function of gamma and eta are shown. We believe that this figure helps to illustrate how the different variables affect the network’s fixed point. However, the effect of $K$ is missing in Fig. 4. To

enhance the understanding of $K$'s impact on the network's fixed point, we have included its effect in Fig. 4.

The “Results” section was revised to be more concise and readable following the reviewer’s suggestions.

## Scientific remarks

**Comment 4**
Regarding the neuronal model : I expect the main impact of ATP deficit, associated with the Na/K-pump, to involve the resting potential (i.e. via \(g_{leak}\)), rather than \(v_r\). Though there can be changes in the reset potential during hypoxia, I am not sure: a) that this is a generic response that has been attributed directly to metabolic constraints, nor b) that it does not occur together with or later than the depolarization and with less influence on the neuronal activity (see e.g. [Müller2000] and [Chen2017]).
Of course, as the authors propose a phenomenological model, **it can be fine to achieve a desired type of response to energy availability via a mechanism that differs from the biological one, but this should be explicitly mentioned and justified.** This discussion was not present in the original article for the model; it would be good to have it now.

[Müller2000]:
https://www.sciencedirect.com/science/article/pii/S0306452200000257?via%3Dihub#FIG1
[Chen2017]:
https://www.sciencedirect.com/science/article/pii/S0014299916307890?via%3Dihub#f0010

**Answer**
We agree with the reviewer that the phenomenological behavior could be achieved using different mechanisms. We sincerely believe that the explanation of the mechanisms underlying the EDLIF behavior are clearly explained in the manuscript. However, we have included a paragraph in the discussion mentioning the distinction between EDLIF and other simple energy-dependent models, explicitly mentioning that they use different mechanism to achieve the same desired response (depolarization of the membrane voltage as a consequence of a decrease in available energy and, therefore, an inhibition of the Na/K pump function)

**Comment 5**
Similarly, the choice and relevance of an all-to-all network and an EI-balanced model should be discussed. Was this done purely out of convenience in terms of mathematical analysis or in terms of the type of brain regions and metabolic issues that the authors want to model? This is especially important as EI dynamics (notably asynchronous irregular, which is usually the one chosen for such networks) has very specific properties that have strong implications in terms of decorrelating the single cell properties and the network dynamics (see e.g. [Fardet2020]).
On the other hand, multiple types of neurodegenerative diseases are known to be associated with rhythmic activities with properties that differ drastically from the EI balanced model.

[Fardet2020]: https://journals.plos.org/ploscompbiol/article?id=10.1371/journal.pcbi.1008503

**Answer**
We agree with the reviewer that this might seem like an arbitrary and rather inconvenient decision. There are several reasons why we have taken this decision. From a biological perspective, a metabolic view of neural network activity is easier to assess in vitro using multielectrode arrays. This method allows the use of microscopy and electrophysiological approaches, unifying metabolic markers with electrophysiology. In these schemes, it is reasonable to suppose that neurons present the possibility of a saturated network with an all-to-all connectivity. Critically, our team is currently working on this front, and we look forward to contrasting our *in silico* results with *in vitro* results. Sadly, *in vitro* experiments take longer, and there are several hardware development challenges to be assessed first. We currently don't have those results. However, we would like to illustrate that the decision is aligned with a research line that will produce future evidence, potentially detecting the caveats and differences with *in vitro* data under similar conditions. Also, it is more likely to find future data to contrast the model considering *in vitro* rather than *in vivo* experiments.

We agree that this implies limitation on their validity for *in vivo* evidence contrast. As such we have extended the reasons given to this decision and contextualizing in a scope of *in vitro* experiments. Also, we have made efforts to allow our model to work with more complex architectures. The text has been changed to address this weakness.

Regarding the E-I balanced network, it is important to mention that our network does not show E-I balance in the classical sense, where strong feedback inhibitory weight maintains the E-I balance in a network without plasticity (van Vreeswijk & Sompolinsky, 1996, van Vreeswijk & Sompolinsky, 1998, Renart et al., 2010). Our networks achieve E-I balance through synaptic modifications, particularly by means of the ED-STDP plasticity rule, which drives the network towards a fixed point. However, the notion of E/I balanced network where the balance is achieved by synaptic modifications is less common (Ahmadian & Miller 2021, but see also Hennequin et al., 2017 and Froemke et al., 2015 for work studying E-I balance through synaptic modifications). That's why we believe the term "E-I balanced network" could be misleading in some parts of the text, so we have modified it accordingly for more clarity.

**Comment 6**
In the current state of the article, it is also unclear how important the energy in the neuronal model is (compared to the synaptic constraints) for shaping the network dynamics.
This is especially problematic since the biologically-relevant range for the parameters is not discussed and the combination of values in Figure 12 do not seem to be the most relevant for that discussion.

**Answer**
Regarding the impact of neuronal sensitivity (gamma) and synaptic sensitivity (eta) to energy imbalances, we found that eta has a greater influence on the network dynamics and energy equilibrium point. In fact, gamma does not affect the energy equilibrium point (see Eqn. 33).

In the discussion, we state that the impact of parameter eta (synaptic sensitivity to energy imbalances) is greater than that of gamma (neuronal sensitivity to energy imbalances). While gamma alters the activation functions, the energy equilibrium point is determined by eta. If all

other factors remain constant, a change in gamma results in adjustments to the specific weights and firing rates to achieve the same energy equilibrium (Fig. 7, new Fig. 4). This is illustrated in Fig. 7, where varying gamma does not change the energy equilibrium point (panel (a)), but does alter the specific firing rates and weight values (panels (c) and (d)) to reach that equilibrium point. Conversely, changing only the synaptic sensitivity to energy imbalances (eta) results in changes to both the energy equilibrium point and the weights and firing rates (Fig. 6). This demonstrates that the eta parameter has a greater impact on network dynamics and the fixed point compared to gamma.

The discussion, particularly the section “network dynamics under metabolic constraint,” has been updated to convey this message clearly.

**Comment 7**
> Despite the significance of this relationship, specific details concerning the impact
> of metabolism on neuronal dynamics and neural network architecture remain elusive,
> creating a notable gap in the existing literature.

There are quite a few publications on this topic, particularly in the theoretical literature, that are not cited in the paper.
This sentence, and the absence of mention of these other studies, may give a distorted view of the current state of the art.

**Answer**
Thanks for pointing out the gap in our references on the impact of metabolism on neuronal dynamics and network architecture. We agree that there are several theoretical studies on this topic that we missed citing. We've now added more references to give a fuller picture of the current research and better reflect the state of the art.

**Comment 8**
> electric activity corresponds to 92% of neuron expenses [2]

I'm not sure what part of Jolivet et al. supports that claim. In addition, "electric activity" is unclear in that context and should be replaced with something clearer.
As per Figure 2.C in [2], signaling-related energy usage is around 60% in neurons.

**Answer**
We appreciate the comment, this was a mistake. We have now updated to the up to 75% estimation leaving the Attwell 2001 and Harris 2012 estimations as reference.

**Comment 9**
> Basically, realizing how crucial energy management is for shaping neural systems

That first part of the sentence should be removed.

**Answer**
This part of the sentence was removed.

**Comment 10**

> This study aims to advance our understanding of how local metabolic constraints
> influence the structure and activity of spiking neural networks. To achieve this, we
> introduce a novel energy-dependent plasticity rule and investigate the impact of local
> metabolic constraints through both analytical methods and numerical simulations.

This should either be moved up right after the sentences on EHP (and modified to fit within the 2nd-to-last paragraph) or removed altogether.

**Answer**
Given that the reviewer has not given any concrete reason to perform such change, and that we disagree on the suggestion, we have not made the requested change. The aim of the studies is usually left by the end of the introduction, as we did it. We also think that the objective is properly described. Therefore we were not able to understand the rationale of the suggested change.

**Comment 11**
> Eq. 1

$A_H$ and $A(t)$ should be defined (homeostatic and time-dependent ATP levels).

**Answer**
Thanks for the input, the text was corrected according to the suggestion.

**Comment 12**
> Figure 1

>Why aren't the firing rates in the simulations following the precise values given by the mathematical formula? Is it due to the timestep of the simulation, the number of spikes considered or some other imprecision? This should be discussed or fixed. In particular, the blue and indigo curves should, in theory, not cross in the inset.

**Answer**
The mathematical formula in Eqn. 5 shows the firing rate as a function of the available energy for constant available energy A, this formula does not express how the firing rate changes as a function of available energy and time. In the simulations shown in Fig. 1, the neuron's available energy changes through the simulation and, therefore, the curves in Fig. 1 do not match the mathematical formula in Eqn. 5. This distinction has been clarified in the main text.

In addition, to show more clearly the impact of different gamma energy sensitivities in the neuron activation function, we update Fig. 1 using $\gamma$ = 0, 25, 50 and 100.

**Comment 13**
> Eq. 5

$\tau_{ref}$ should be defined in the text (refractory time); it might also be good to re-explicitate that the dependency in A comes from $s_0$ (thus making it obvious that $\tau_{ref}$ is a constant).

**Answer**
$\tau_{ref}$ is defined in the text. Eqn. 3 explicitly shows the depende of the voltage membrane through the reset potential. In addition, Eqn. 4 shows that the interspike interval is a function of the available energy, where the reset potential changes depending on the available energy. In Eqn. 5 we include an explicit depende of the available energy for the interspike interval, making clear that the firing rate dependency on available energy is related to the interspike interval dependency in available energy, and not in the refractoriness of the neuron.

**Comment 14**
>Eq. 17

\(\lambda\) was already used as the firing rate in Eq. 5, please change one of the two for consistency.

**Answer**
The text has been appropriately rectified as per your suggestion.  Now we use $\nu$ for the firing-rate.

**Comment 15**
> Eq. 18

\(\alpha\) was already defined as \(v_{rest} /v_{th} − 1\) in Eq. 2, please change one of the two for consistency.

**Answer**
Thanks for the suggestion, the text has been duly corrected. Now $v_{rest} /v_{th} − 1 =  \ro$

**Comment 16**
> There is biological evidence suggesting the existence of excitatory-inhibitory (E-I)
> balanced networks in different parts of the brain, such as the CA3 region of the
> hippocampus, basal ganglia, and the primary visual cortex [27]

Citation [27] is a simulation study, please provide direct citations of biological papers asserting the relevance of balanced networks in each of these three regions (especially basal ganglia).

**Answer**
Thank you very much for detecting this problem. We have now updated using a set of experimental references which estimated the ratio of E-I 70-30 for the cortex.

**Comment 17**
> Because both LTP and LTD depended on the activation of NMDA receptors and are absent
> in cases in which the postsynaptic neurons were GABAergic in nature [28], only
> excitatory-excitatory connections are plastic in our E-I network.

The plasticity of excitatory synapses on inhibitory postsynaptic neurons is a well-known phenomenon ([Malenka2004], [Lu2007]). It has also been demonstrated that many types of synapses can display LTP, including inhibitory ones ([Haas2006]).

[Malenka2004]: https://www.cell.com/neuron/fulltext/S0896-6273(04)00608-7
[Lu2007]: https://pubmed.ncbi.nlm.nih.gov/17804631
[Haas2006]: https://doi.org/10.1152/jn.00551.2006

**Answer**
Indeed, as the reviewer suggests there are some well understood mechanisms of plasticity in excitatory and inhibitory neurons. It is relevant to notice that LTP or LTD are not the only plasticity mechanisms described (Fields, 2015, Barral et al., 2016, Fong et al., 2015). They are the best understood, especially for excitatory neurons. We know that LTP and LTD are present in inhibitory neurons. We did not mention such on the draft. The reason has to do with cellular physiology.

Firstly, we would like to address that there are not inhibitory synapses, rather inhibitory neurons. This is relevant, as they are two different kinds of neurons which present differences in their cellular and molecular physiology. The references given by the reviewer are consistent with more recent evidence supporting that LTP in inhibitory neurons is common (Caponga et al., 2020). However, the mechanisms are highly heterogeneous (Caponga et al., 2020), and they don't necessarily display an STDP-like scheme. To our knowledge, there is no evidence that those mechanisms are directly modulated by energy availability, as those reported in excitatory neurons (Potter et al., 2010).

Given these differences, we chose to fix all synapses except the excitatory-excitatory ones. Inhibitory neurons are not as homogeneous in their synapses as excitatory neurons, making it harder to generalize global mechanisms. We believe this approach is best for an initial exploration of the energy dependence of plasticity. Nonetheless, we acknowledge the importance of inhibitory neurons. Our model, which is openly available, can integrate plasticity rules for inhibitory neurons, and we plan to explore these elements in future studies.

We agree that our initial wording was unclear, so we have revised the sentence to:

"Because both LTP and LTD depended on the activation of NMDA receptors and are absent in cases in which the postsynaptic neurons are GABAergic [34] and that LTP and LTD mechanisms in inhibitory neurons are widely diverse on their physiology [35], only excitatory-excitatory connections are plastic in our E-I network. The main reason for this is that we cannot generalize the energy dependence observed for excitatory neurons, given that inhibitory neurons have relevant physiological differences compared to excitatory neurons."

**Comment 18**
> To numerically test our theoretical predictions, the network is simulated utilizing the
> Neural Simulation Tool program (NEST) [29]

Please cite the specific version of NEST that was used:

https://nest-simulator.readthedocs.io/en/stable/citing-nest.html
The Scholarpedia article is not a relevant citation for software.

**Answer**
The reference has been updated. For the study we used NEST version 3.3.

**Comment 19**
> Figures 14C/15C

Do not use the average value in 15C but a 2D distribution. I would actually suggest using a distribution for Figures 8, 10, 13, and 15, both for consistency and because some other plots have values that are not completely stationary.

**Answer**
We appreciate the reviewer's suggestion. We've updated all the figures showing simulation results. Specifically, instead of plotting the mean and standard deviation of available energy for the excitatory population, we now display the available energy per neuron in each experiment (panel (a) in Figs. 5, 6, 7, and 8), making the division of the excitatory population into subpopulations more clearly visible.

However, for the previous Figs. 8, 10, 13, and 15 (panels (b) and (c) in new Fig. 5, and panels (c) and (d) in new Figs. 6, 7, and 8), we decided to keep the original representation instead of using distributions. The main reason is that plotting each neuron in the network allows us to show the average available energy for that neuron, which is important information that might be hard to convey with distributions alone. We believe this approach gives a clearer understanding of the energy dynamics within the network.

**Comment 20**
> In addition, following in vitro measured weights strength in neuronal cell
> assemblies [32], initial synaptic strength values for the simulated network are drawn
> from an exponential distribution

Regarding network properties ([Barral2016]) or similar experimental works in vitro or in vivo would be more relevant given the specific model studied.

[Barral2016]: https://www.nature.com/articles/nn.4415

**Answer**
This is indeed an exciting direction for future work. For our current study, we used an exponential distribution for initial synaptic strengths, which has been effective for our exploration. The generality of our findings regarding the fixed point analysis for the networks is not restricted to the exponential distribution. We believe our current approach provides valuable insights, but we agree that incorporating data from studies like Barral et al., 2016, could enhance the relevance and accuracy of future models.

**Comment 21**
> Eq. 40

This does not seem correct, unless I misunderstood how the matrices are constructed (in

which case it should be explained in more details).

From what I understand, Eq. 39 is for a single neuron (let's call it \(i$). It could (should ?) actually be written:

A¯ti=AHi-1KEapv¯ti+Esyn∑jw¯tijv¯tj \overline{A}^i_t = A^i_H - \frac{1}{K} \left[ E_{ap}\overline{\nu}^i_t + E_{syn} \sum_j \overline{w}^{ij}_t \overline{\nu}^j_t \right]

In that perspective, we can write the vector of activity on the set of all neurons \(i \in [1, 500]\), i.e. \(\overline{\mathbf{A}}_t = \{ A^i_t \}_i \).

The only matrix involved is \(\overline{\mathbf{w}} = \{\overline{w}^{ij}_t\}_{i, j} \) and the whole equation becomes:

A¯t=AH-1KEapv¯t+Esynw¯tv¯t \overline{\mathbf{A}}_t = \mathbf{A}_H - \frac{1}{K} \left[ E_{ap}\overline{\boldsymbol{\nu}}_t + E_{syn} \overline{\mathbf{w}}_t \overline{\boldsymbol{\nu}}_t \right]

I.e.

A¯t=AH-1KEap+Esynw¯tv¯t \overline{\mathbf{A}}_t = \mathbf{A}_H - \frac{1}{K} \left[ E_{ap} \mathbb{I} + E_{syn} \overline{\mathbf{w}}_t \right] \overline{\boldsymbol{\nu}}_t

Or, if one wants to transpose everything,

A¯t⊤=AH⊤-1Kv¯t⊤Eap+Esynw¯t⊤ \overline{\mathbf{A}}_t^\top = \mathbf{A}_H^\top - \frac{1}{K} \overline{\boldsymbol{\nu}}_t^\top \left[ E_{ap} \mathbb{I} + E_{syn} \overline{\mathbf{w}}_t^\top \right]

**Answer**
The reason for the confusion lies in the construction of the matrix w. In the network model section, the creation of the weight matrix is described. In particular, we say “where the i, j element of w correspond to the synapse from the ith presynaptic neuron to the jth postsynaptic neuron.” In general, the i,j element of w corresponds to the synapses from the jth to the ith neuron. Hence, you arrived at a different expression.

If $w$ is constructed such that the $i,j$ element correspond to the synapses from jth to ith neuron, then the expression you derived is correct. This means that the weight matrix used in your derivation is the transpose of the weight matrix we used in our original derivation.

However, we agree that the construction of the weight matrix is not practical. Therefore, we decided to align with the standard way of doing it. Now, the i,j element in the weight matrix describes the connection from the jth to the ith neuron. All the matrix equations in the manuscript have been rewritten and, in particular, previous Eqn. 40 (new Eqn. 39) aligns with the reviewers proposal.

**Comment 22**
> it is possible to neglect the neuron's own action potentials energy consumption

This is indeed the case with the values of \(E_{ap}\) and \(E_{syn}\). However, it seems to directly contradict the results from [2] and ([Howarth2012]) where \(E_{ap} / E_{syn}\) is 20%/60%.
As the values of \(E_{ap}\) and \(E_{syn}\) are currently not justified, it seems absolutely necessary to explain why these specific values were chosen.

[Howarth2012]: http://journals.sagepub.com/doi/10.1038/jcbfm.2012.35

**Answer**
This is a very important point that we didn't mention in the article but is absolutely necessary to include.

We can estimate the average energy consumption in the neuron for action potentials ($C_{ap}$) and postsynaptic potentials ($C_{syn}$) as follows:
- $C_{ap} \approx \nu  E_{ap}$
- $C_{syn} \approx \nu  E_{syn} N <\bar{w}>$

where \nu is the average firing rate, N the number of neurons in the network, and $<\bar{w}>$ the average value of the absolute and normalized synaptic strength ($w\w_{max}$, as defined in the manuscript). In our simulations, we initialize the weights following an exponential distribution with scale parameter $\beta = 5$ and $w_max = 100$, so $<w\w_{max}> = 1/20$.

Then, the ratio between $C_{ap}$ and $C_{syn}$ is:
$\frac{C_{ap}}{C_{syn}} = \frac{\nu E_{ap}}{N \nu E_{syn} <\bar{w}>}$

If we force the $C_{ap}/{C_{syn}$ ratio to be equal to $20/60$ and match the results shown in Howarth et al., 2018, we arrive to the following relation:

$E_{ap} = \frac{N <\bar{w}>}{3} E_{syn}$,

where $(N <\bar{w}>)/3 \approx 8.3$ (500/60 given the parameters in our simulations).

In our original simulations $E_{ap} = 4 E_{syn}$. So the chosen values maintain the same order of magnitude that we would obtain following Howarth et al., 2012 results, for the $C_{ap}/C_{syn}$ ratio.

However, we are interested in simulating our networks with variables more representative of biology, so we decided to run all the simulation again using $E_{ap} = 8.2 E_{syn}$, thus aligning our numerical experiment with the energy consumption ratio presented in Howarth et al., 2012.

One additional observation: in our framework the energy fixed point is independent of $E_{ap}$ and $E_{syn}$ (Eqn. 33). Thus, when we simulated the network with a $E_{ap} /E_{syn}$ ratio closer to the one obtained in Howarth et al., 2012 ($E_{ap}/E_{syn} = 8.2 \Rightarrow C_{ap}/C_{syn} \approx 20/60$), the same energy fixed point drives the

network's activity (given by Eqn. 33). However, the weights and rates associated with those simulations slightly vary.

**Comment 23**
> Exploring synaptic energy imbalances sensitivity

These paragraphs are really hard to read, even knowing what is going on. An additional schematics of the transition, rather than lengthy paragraphs, would probably make this much more understandable.

**Answer**
Thank you for the feedback. We understand that the original text was difficult to follow. In response, we have rewritten most of this section to express our message more clearly. Specifically, we have updated the explanation for the appearance of oscillations under small K in our simulations as follows:

“To understand this intuitively, it is useful to remember that neurons with impaired metabolism (small $K$) have slower responses to energy consumption, causing a higher delay between consumption and production. Also, excitatory-excitatory weight changes depend on the available energy in the neurons (see Eq. 32 and Fig. 2).
If we start the simulation with all neurons at the homeostatic energy level $A(t = 0) = A_H$ , and the energy fixed point is lower than the initial energy ($\breve{A} < A_H$), then initially, weights will increase, and firing rates will rise due to the current-rate relationship. This leads to increased energy consumption, but because energy production is delayed, the available energy will tend to decrease ($\Delta A(t) < 0$).
As available energy continues to drop, eventually it will be lower than the energy fixed point ($A(t) < \breve{A}$). When this happens, excitatory-excitatory weights will decrease, reducing firing rates. This process will continue until energy consumption decreases enough for energy production to surpass it. When energy production surpasses consumption, the available energy in neurons starts to increase ($\Delta A(t) > 0$) until it matches the energy equilibrium point ($A(t) = \breve{A}$).
Because energy production is always active below the fixed point ($A_s (t) = K(AH − A(t))$), this allows the available energy to rise above $\breve{A}$. When the available energy exceeds the equilibrium value, the cycle repeats: weights and firing rates increase, leading to higher energy consumption than the neuron can handle, causing another drop in available energy ($\Delta A(t) < 0$).
This cyclical behavior stops when energy production and consumption match, maintaining available energy near the fixed point ($A(t) \approx \breve{A}). Achieving this equilibrium is easier when on-demand energy production can compensate for consumption in neurons.”

**Comment 24**
> Interestingly, when the synapses are highly sensitive to energy
> imbalance (Fig. 10E and 10F), it is not possible for all the neurons
> to achieve the metabolic fixed point, and some neurons achieve
> the silent fixed point previously described.

A more precise discussion of the mathematical transition for silenced neurons would be appreciated.

**Answer**
In principle, the appearance of silent neurons might seem hard to understand, but the mechanism for their emergence is not complicated. We agree that our explanations in the first draft were hard to follow. The updated explanation for the emergence of silent neurons when $\eta$ is too large or K is too small is as follows:

“When $K = 0.5$ (purple in Fig. 8), the mean firing rate and excitatory-excitatory weights decrease compared to the $K = 0.7$ case. However, the network can still converge to a global fixed point if a subpopulation of neurons becomes silent. This behavior aligns with the energy constraints of the network. As the ATP production parameter K decreases, energy consumption must also decrease. To achieve this, excitatory-excitatory weights and mean excitatory firing rates decrease, resulting in a subpopulation of silent neurons to reduce energy consumption.
This phenomenon is similar to what happens when there is high synaptic sensitivity to energy imbalances ($\eta = 100$ in Fig. 7), but the cause is different. With high synaptic energy imbalance sensitivity ($\eta$), the energy level fixed point \breve{A} is close to the homeostatic energy level $A_H$ . This requires a reduction in energy consumption to reach a global fixed point.
In the case of metabolic impairment, the energy level fixed point \breve{A} is not necessarily close to $A_H$. Here, energy consumption needs to drop because impaired energy production cannot meet high energy demands. This balance between energy consumption and production is necessary for the network to reach a fixed point. Therefore, the constraint in the metabolic impairment case is imposed by the neurons’ energy production capability, not by synaptic energy sensitivity. Despite the different causes, the outcome is similar: silent neurons in both scenarios are those with higher inhibitory synapses.
This is because, before the network divides into two subpopulations, an increase in available energy is followed by a decrease in excitatory-excitatory weights. The first neurons to become silent are those with higher inhibitory weights as they receive less incoming excitatory current.”

**Comment 25**
> Fig 9C (and more generally)

Mean and standard deviation are not relevant for a bimodal distribution, please plot each monomodal subpopulation separately.

**Answer**
We have improved all the figures related to numerical simulations. Now, we plot each neuron's available energy throughout the simulation, so no mean and standard deviation are used. Please refer to the answer to comment 19 for more details.

## Technical remarks

**Comment 26**
The provided code contains only the NESTML model ([https://github.com/Wiss/nestml](https://github.com/Wiss/nestml)).
It would be preferable to provide all the relevant code necessary to replicate the result in the study. Furthermore, rather than include the new model within a fork of the nestml repository

(which requires to download 80MB of data to access 2 files of 3kB each), it would be better to include them in a separate repositories.
I suggest uploading all the relevant code in the following form:

```
Jaras_et_al_MetabolicConstraintsModel/
├── README.md # instructions to install the models and run the scripts
├── Models/
│  ├── ed_stdp_synapse.nestml
│  └── third_factor_stdp_synapse.nestml
└── Simulations/
├── fig1.py
├── ...
└── fig16.py
```

This is just an example structure. Some figures may be grouped within a single file and some separate simulation files may be added to separate simulations and analysis whenever relevant.

**Answer**
The referenced link in the comment is not the one we originally provided. The original link is: https://github.com/Wiss/nestml/tree/edlif_plus_tfsr/ehp/network which points to the “edlif_plus_tsfr” branch and the network folder, where all the necessary code is available.

Following the suggestion, the code is now hosted in a new repository: https://github.com/Wiss/edsnn
We also included the GitHub repository link in the manuscript

## Typographic remarks

**Comment 27**
Adapt the size of figures or the figures' fontsize such that the text in the figures appears similar to the main text.

There are issues with quotes. If LaTeX was used, make sure to use ``this" and not "that" for quotes.

**Answer**
We have adjusted the size of the figures and the font size within the figures to match the main text for better consistency. Additionally, we corrected the use of quotes in the manuscript.

**Comment 28**
> where synaptic potentiation and depression balance each other in average.

*on* average

**Answer**
Thanks for the correction. The text has been updated.

**Reviewer #2**:

**Comment 29**
In this paper the authors sought to explore the effects of modifying a spike-timing-dependent plasticity rule to account for energy constraints on a neuron. They analyzed in particular a spiking neural network's ability to attain a fixed mean firing rate as a function of various changes to the metabolic parametrization. They further considered pathological effects on a fixed firing rate due to metabolic limitations. I agree with the authors that energetics are not (to my knowledge) considered in most neural network studies, and in that regard this work could fill a gap in the literature. However, a number of concerns and limitations in the present work dampen my enthusiasm.

**Answer**
We thank the reviewer for the comment.

**Comment 30**
1. The authors claim to explore E/I balanced networks yet neglect an entire field's worth of work on the subject (see, e.g. van Vreeswijk, Sompolinsky, Renart, Doiron) demonstrating a rich variety of dynamics which could have been explored. Nor do the authors defend this choice through an appeal to other work which discusses different notions of balance (e.g. Ahmadian & Miller 2021). Figure 3 does not add much to the story outside of illustrating parameters. Moreover, these weight parameters which are critical in appreciating balance and the dynamical regime of the network are nowhere provided (and not included in Table 1). It would be important to include the chosen w_ij values, and the range of w_ee, or max(w_ee) (this I attempted to infer from later plots to be O(1)). Additionally Figure 4 is at best not helpful and at worst misleading because the network is stated to be all-to-all connected and no concept of distance-dependent connectivity or weight modulation is mentioned, so the spatial distribution of the neurons on some grid is irrelevant. Further, balanced regimes can lead to chaotic dynamics. While the firing rate plots demonstrate this is not the case here, all of the math related to a fixed point solution depends on assumptions about the dynamical regime of the network which is not stated.

**Answer**
An important difference between our work and previous work referenced by the reviewer, is that, to the best of our knowledge, the cited work is limited to the exploration of E/I balanced networks without synaptic plasticity (e.g., van Vreeswijk & Sompolinsky, 1996, van Vreeswijk & Sompolinsky, 1998, Renart et al., 2010). As mentioned in Ahmadian & Miller, (2021), excitation and inhibition can be balanced in at least two ways. The reviewer's references follow the strong-inhibition and fixed weights approach. The other approach consists of achieving a balance between excitatory and inhibitory activity through synaptic modification, where synaptic plasticity rules help to achieve the balance. Our work follows the second approach. An important contribution of our work is the inclusion of energy-dependent synaptic plasticity to explore the dynamics of spiking neural networks under metabolic constraints. This is why we focused on the network's dynamics under different sensitivities to energy imbalances at the neuron and synaptic level, instead of exploring different dynamics

shown in the cited articles. Additional references regarding E-I balanced networks through synaptic modifications were included in the text.

We agree with the reviewer that Fig. 4 did not add relevant information, so we decided to remove it.

The weights are sampled from an exponential distribution described by Eqn. 28. We forgot to include the chosen scale parameter for the weight distribution. We have updated the manuscript to include the value for the scale parameter $\beta=5$.

Regarding the assumptions to achieve the fixed point:

- The synaptic plasticity fixed point relies on a symmetrical distribution for pre- and postsynaptic spiking times (mentioned in the text)
- The possibility of achieving the network's fixed point depends on the ability of neuronal energy production to compensate for energy consumption. Otherwise, (K too small) it is not possible to achieve the fixed point. Our simulations also support this claim. For a detailed list of specific assumptions and approximations made while deriving the network's fixed point, please see the answer to the next comment.

**Comment 31**

2. I am confused about the author's approach in comparing theory and simulations. Much biological richness was omitted in choosing homogeneous network parameters. More complete analysis could perhaps have been achieved with a smaller, simplified architecture (e.g. two E neurons). Further, the authors comment on numerous occasions that certain approximations hold in a large-N limit (e.g. pg 16 last paragraph). In this vein, no attempt to match theory and simulation outside of qualitative discussion was attempted for e.g. figures 10, 13, 15 which seem to permit such a representation. Further, the figures in which some theoretical-type estimate was included didn't have any mention of why the sims would (and often do) disagree.

**Answer**

In our opinion, two-neuron simulations omit considerably more biological richness than a homogeneous network. A large amount of biology research is conducted in *in vitro* neural cultures that are fairly homogeneous in their connectivity (although they can be quite heterogeneous in other aspects). We selected this approach as we are working on obtaining *in vitro* results to contrast with our *in silico* model and because it is easier to find metabolic-related data. The experiments presented are also aligned with experiments that can be carried out in *in vitro* setups. We realize that this may depart from a more classical approach, however, for us, it is critical to evaluate these predictions empirically in the near future.

Additionally, experiments with *in vitro* behavioral paradigms such as those presented by Novellino (2007), Tessadori (2012, 2013), and Kagan (2022), show how even in these experimental set ups where architecture has been lost, it is still possible to teach a task to a biological neural network. Given that this is a more accessible experimental setup, it would be easier to contrast our *in silico* findings.

We agree that these explanations were not explicitly addressed during the decisions or even in the discussion. We have improved this throughout the draft.

Regarding the theoretical approximations in Figs. 9, 12, 14, and 16, in principle, the theoretical energy equilibrium follows Eqn. 33. However, when exploring the energy dynamics and fixed point, several approximations and assumptions were made:

1. To simplify the mathematical tractability, in Eqn. 36 we use the delta kernel for $\epsilon_{ap}$ and $\epsilon_{syn}$, whereas in the simulation we have exponential kernels.
2. From Eqns. 36 to 37, we assume time scales separability between learning and spike dynamics.
3. In Eqn. 38, we find a relation that holds on average (the subindex “t” means that we are taking the average of that variable in a time window from t to t + T).
4. In Eqn. 40, we assumed that the time-averaged available energy approximates the energy equilibrium given by Eqn. 33 (needed for achieving the weight’s fixed point).

Which finally leads us to Eqn. 41: the best approximation we found to estimate the energy consumption at the fixed point. Given the necessary assumptions and approximations to obtain Eqn. 41, we do not expect to perfectly  match the average available energy in the network simulations. However, we think that the approximations are good enough, as can be seen in Figs. 6, 7, 8 and 9, and provides a deeper understanding of energy dynamics in spiking neural networks compared to the current knowledge in the field.

To provide more evidence regarding our theoretical approximation, we simulated a simplified scenario containing excitatory neurons. In the experiment, there are 1000 presynaptic neurons bombarding only one postsynaptic neuron. We use the ED-STDP rule and the initial value for all weights is zero. As $\eta$ increases, $\breve{A}$ gets closer to $A_H$, in line with Eqn. 33. Our prediction (gray dashed line) aligns well with the numerical results. The code for this experiment could be found at
https://github.com/Wiss/edsnn/blob/main/edstdp/edstdp_diff_eta_sensitivity-bombard.ipynb

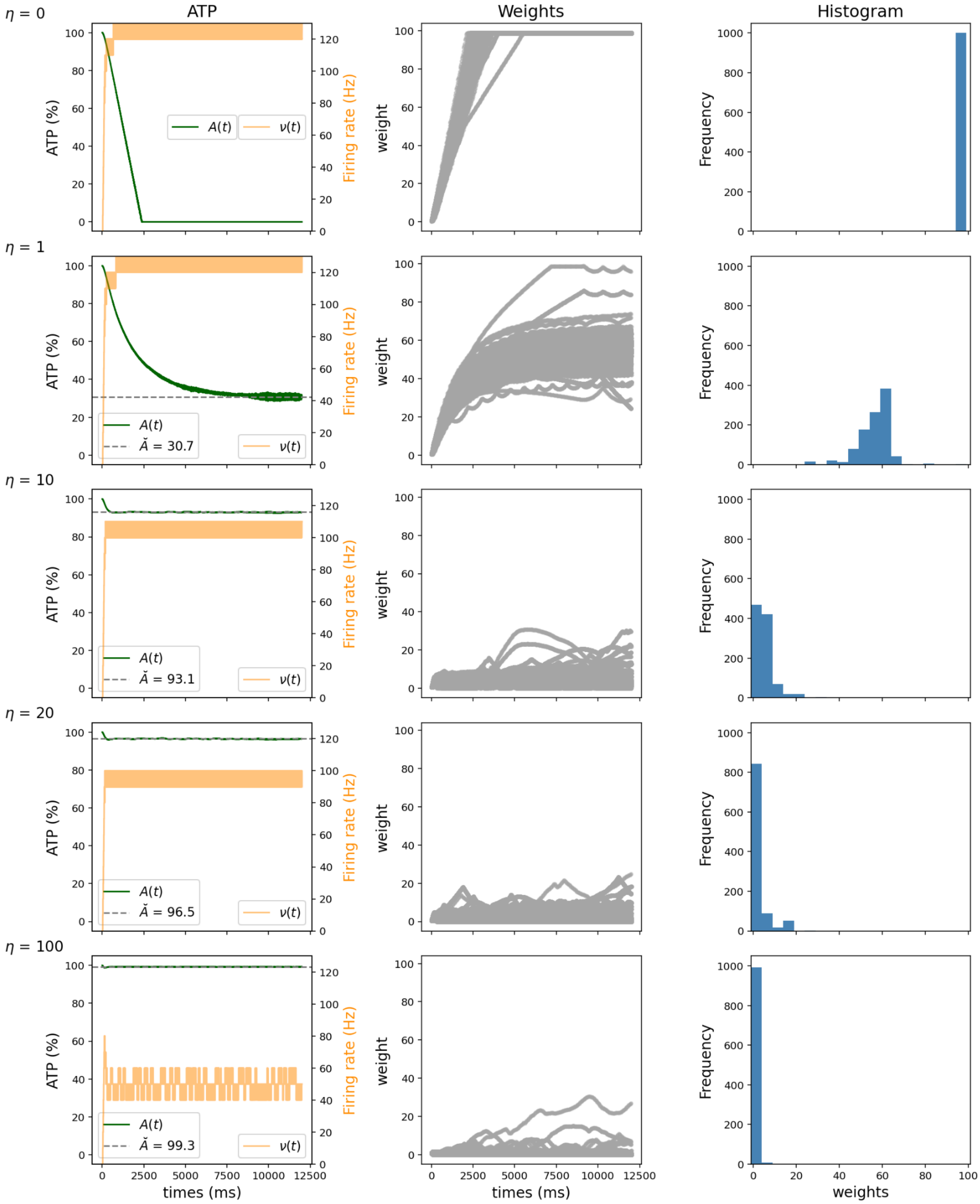


**Comment 32**

3. In the Discussion, I think the suggestion of therapeutic relevance is a bit far-fetched. I'm also not convinced that the suppression of a subset of the neuronal activity through what I understand to be disconnection (or extremely low E-E connections) is consistent with the

idea of sparsely-firing neurons in cortex, as suggested in the discussion. More would need to be said/done to further this case.

**Answer**
We regret that the reviewer was not convinced. However, we have molecular biologists working with us who disagree. Currently, neural pharmacology has two approaches: modulating activity through neurotransmission (which can be reinterpreted as modulating energy expenditure), and more recent drugs focused directly on mitochondrial energy production (Cenini et al., 2019).  There are several fine-tuning mechanisms for energy management in neural tissue. Involving glia (Weber et al., 2015), and as we proposed from the results of (Potter et al., 2010), synaptic weight. This evidence is also supported by synaptic scaling protocols where changes in activity are driven by synaptic weight scaling (Turrigiano, 2008, Turrigiano, 2011). All this was explained in the discussion section. The reviewer may disagree, however considering it far-fetched may only reflect a traditional view of neural physiology, which has limited us to develop fairly the same therapeutic strategies for over four decades.

Regarding the suppression of a subset of neuronal activity to meet metabolic constraints, in the discussion we hypothesized that in the brain (where we have a high number of neurons), local energy constraints are enough to show that some neurons will remain silent if the network achieves a network energy equilibrium. Otherwise, energy consumption is too high to be compensated by energy production in the neurons. Given our theoretical framework and simulations, this hypothesis aligns with our observation. Therefore, we do not understand the potential inconsistency mentioned by the reviewer in our hypothesis.

To be completely clear here. The silenced neurons do not necessarily have weak outgoing E-E connections, otherwise their activity would barely affect postsynaptic neuron activity. Therefore, while the network tries to settle at a fixed point, there is a global process of decreasing excitatory activity (through decreasing E-E connections). The neurons with higher incoming inhibitory activity, will be the first in becoming silent neurons. Once they are silenced, their incoming E-E connections stay fixed (no spikes to update the E-E connections), while the activity of other non-silent neurons would adapt until reaching the network's fixed point. In this sense, silent neurons allow postsynaptic neurons to settle and, as a consequence, they allow the network to achieve a fixed point.

**Comment 33**
4. The network effects seem rather insensitive to choice of \gamma (Figs 1 and 9). Also, no discussion on the relevant order of certain parameters was attempted. This could help mathematically, but in addition the authors claimed to capture existing results from data yet this does not seem to be seriously considered without such a discussion.

**Answer**
A deeper exploration of the impact of \(\gamma\) at the neuron level is given in Jaras et al., 2021. In that study, the model was also tested using real data.

Regarding the impact of the neuronal sensitivity ($\gamma$) and the synaptic sensitivity ($\eta$) to energy imbalances, eta has a greater impact in the network dynamics and energy equilibrium point. In fact, gamma does not affect the energy equilibrium point (see Eqn. 33). However, because gamma modifies the neuron’s activation function, if everything remains

equal, different values of gamma will induce different weights and firing rates combinations to achieve the same energy equilibrium (previous Fig. 7, new Fig. 4). This is shown in Fig. 7, where different values of gamma do not change the energy equilibrium point (panel (a)), while the specific firing rates and weight values change (panels (b) and (c)) to achieve that equilibrium point. On the other hand, if we only change the synaptic sensitivity to energy imbalances ($\eta$), then the energy equilibrium point as well as the weights and firing rates changes (Fig. 6), this shows that eta parameter has a greater impact on networks dynamics and fixed point compared to gamma. Results and Discussion sections have been updated to convey this message more clearly.

We claimed to qualitatively capture phenomenological behavior observed in biology. In particular, our ED-STDP plasticity rule replicates the suppression of long-term potentiation when the available energy on the postsynaptic neurons drops (Potter et al., 2010). However, there is no data to our knowledge that combines in the same recording metabolic and electrophysiological data (a more detailed explanation is given in comment 38). This is one of the reasons why we didn't tune our model with real data. However, we have updated all the simulations in the manuscript using new values for synaptic energy consumption ($E_{syn}$) and action potential energy consumption ($E_{ap}$) to be consistent with the energy expenditure ratios published in Howarth et al., 2012. We also revised the discussion section to clarify these points.

**Comment 34**
5. The only really interesting dynamics appear only in the (apparently) pathological case of K very small (Fig. 14). What happens in the case of heterogeneous networks? Currently the only source of heterogeneity is in the input.

**Answer**
Even though the majority of the simulations end in a (previously unknown) stable fixed point, we think this behavior is very interesting, given that simulations with a network with the same parameters, but without energy constraints, explode (neuronal activity saturates, as in Fig. 8 of the original manuscript, new Fig. 5). One of the main points our work shows is that the inclusion of energy-constraints drives the networks towards different fixed points, and that fixed point depends on the sensitivity of the synaptic plasticity ($\eta$) and neuron ($\gamma$) to energy imbalances.

At the moment, the only source of heterogeneity is the input to the neurons and weights. Like the reviewer, we also think that a general framework accounting for more heterogeneity would be very valuable for the field. However, we think the homogeneous case is a good starting point and provides interesting insight at the network level. In addition, we would not support the idea of showing heterogeneous simulations without developing a deeper analytical understanding first, so we can interpret what is happening instead of only trying to observe more diverse behavior at the network level.

**Comment 35**
6. K is overloaded as a parameter in the text (eqs 7 and 17).

**Answer**
Thanks for your input. The temporal filter in Eqn. 17 is now represented by the Greek letter

kappa.

**Comment 36**
Additional points:
1. Eq 1, I(t) is not defined. Should be stated.
**A:** Following the suggestions, we defined $I(t)$ after Eqn. 1

2. Eq 2, A_H is not clearly defined until later, and should be discussed at least within the same section.
**A:** We defined $A_H$ after Eqn. 2

3. Between eq's 10 and 11; 12 and 13 it is unclear why a Heaviside is introduced when the integral over epsilon is already bound to 1, and then an exponential function is later used for epsilon. It seems to add a number of equations and text which seem to be unnecessary.
**A:** If the Heaviside function is not introduced, then the neuron response is not causal anymore.
For example, using Eqn. 10 and an exponential kernel:
$A_{syn}(t) =E_{syn }\bar{w} e^{-(t-t_{s})/\tau_{syn}^{A, k}},$
$A_{syn} at time zero would be affected by a future spike activity ($t_s > 0$), because nothing impedes evaluating the exponential kernel with $t < t_s$. On the contrary, this anticausal behavior could be avoided by including a Heaviside function $\Theta(t-t_{s})$, such that future activity does not affect current metabolic state of the neuron, i.e., only allowing causal metabolic response in the neuron.
However, we agreed with the reviewer regarding the exclusion of the Heaviside function when showing that \varepsilon is bounded ($\int_{0}^{\infty}\varepsilon(t)dt = 1$). That Heaviside function is only needed if we integrate from minus infinity to infinity, but because we are integrating from 0 to \infty, the Heaviside function is not needed, so we took it out.

4. Following Eq 20, it was stated that Eq 20 was left as-is for future general readers while a specific case is examined here which dramatically simplifies the $\Delta w$ equation. Considering a general form is already provided in eq 17, it would be nicer to simply write the specific case examined here in place of the current Eq 20.
**A:** Eqn. 17 shows the general form for STDP plasticity without energy dependency, while Eqn. 20 presents our proposal for the general form including energy dependency in LTP and allowing to tune $\mu_{\pm}$ to create multiplicative or additive learning rules accounting for neuronal metabolic dependencies. Then we use Eqn. 20 to define an additive learning rule ($\mu_{\pm} = 0$), which allows us to mathematically explore the impact of energy-dependent STDP in a network. We decided to keep Eqn. 20 as it is, because we think it might be valuable for readers searching for more general energy-dependent STDP rules, beyond the general form presented in Eqn. 17, where no energy dependencies are present.

5. Should eq 27 not be a strict equality in the linear case? What is approximated?
**A:** Even though in Eqn. 27 the activation function \phi is linear, the approximation appears because we are approximating the firing rate of the neuron in a time window using the mean incoming current to the neuron in that time window ($\mathbf{\bar{\nu}_{t}^{\top}} \approx \phi(\mathbf{\bar{I}_{t}^{\top}})$). The fact that we are using the mean incoming current for

calculating the firing rate, implies that we always get an approximation for the firing rate term independent of whether \phi is linear or not.

6. The extensive discussion of trivial solutions (e.g. pg 16 of the manuscript) detracts more than it adds from the exposition.
**A:** We agreed and took out trivial solutions' explanations.

7. Eq 4 V_th should be lower-case
**A:** Eqn. 4 has been corrected following the suggestions.

8. Figure 11 does not illustrate the two subpopulations, simply shows a separation in the ATP dynamics. Would be better to show the actual network responses as well.
**A:** The results previously shown in Fig. 11 are now illustrated in Fig. 6. For $\eta = 100$ (red), it is indeed possible to observe a separation in ATP dynamics corresponding to the two subpopulations, which can be distinguished in panels (c) and (d). Specifically, in panel (c), it can be seen that one population does not present spiking activity.

9. The notation for total presynaptic input is awkward and should be reconsidered (i.e. superscript ex \to ex, k is very cumbersome)
**A:** Thanks for the suggestion. We updated the notation according to the reviewer's suggestions.

**Reviewer #3**

**Comment 37**
This study presents an energy-dependent STDP plasticity rule combined with a previously established energy-dependent LIF model to investigate the effects of metabolic conditions on firing in a spiking neural network model. The strength of the study is its focus on the understudied topic of energetics in neural network models as well as its employment of both analytical and numerical methods. Their simulations predict network activity affected by metabolic imbalances and disruptions.

**Answer**
We thank the reviewer.

**Comment 38**
This is a potentially highly interesting energy-dependent model of synaptic plasticity. **However, the authors do not show how close it is to real neurons and synapses in terms of the time scales of ATP and synaptic changes**. The authors have not tuned the model to experimental data. They only claim that the model replicates the "suppression of long-term potentiation when the available energy level on the postsynaptic neuron drops". It would be important to show that the simulated weight changes, FR changes and energy expenditure/supply changes (ideally under different metabolic conditions) correspond to some measured weight and FR (and ideally also ATP) changes. Models published in Plos CB are usually closely linked to experimental data.

In summary, the main issue is the lack of links to data to tune or justify the models.

**Answer**
We agree with the reviewer. However, there is no data to our knowledge which combines in the same recording metabolic and electrophysiological data. This is not a minor technical issue. On the one hand, that needs the co-recording of microscopy and electrodes. You may do this up to some regard *in vivo*, but microscopy common probes are more related to activity than metabolism, giving similar information to tetrodes. *In vitro*, there is a wider range of metabolic probes, however, the neural culture lasts about an hour or less out of the incubator and the electrodes occlude light. Without even considering obstacles such as multielectrode array size is not compatible with most of the best microscopes, and that most amplifiers of multielectrode arrays heat themselves up to a dangerous point for neural culture viability. The other challenge is that temporal resolution of microscopy is in the order of seconds. However, electrophysiology occurs in ms, meaning a major difficulty in synchronization. But above all, it is not possible with current technology to follow metabolic dynamics in the order of ms.

As such, we definitely agree with the reviewer, and we will make our best effort to be the first on reporting such data. We are currently working on replicating these *in silico* experiments *in vitro*. But obtaining such data will be demanding as we are developing *ad hoc* hardware to do so. We have extended our limitations to incorporate this comment.

However, we are interested in simulating our networks with variables more representative of biology, so we decided to run all the simulation again using $E_{ap} = 8.2 E_{syn}$, thus aligning our numerical experiment with the energy consumption ratio presented in Howarth et al., 2012.

**Comment 39**
* The authors should clarify which experimental data justify that the "energy-dependent STDP rule uses postsynaptic available energy as a neuromodulator, resulting in the suppression of LTP and enhancement of LTD at low postsynaptic energy levels (Fig. 5)

**Answer**
We do present evidence to support the modification to the STDP rule. “Our energy dependent plasticity rule is inspired by the findings shown in [26].”

We agree that this sentence should be extended for better comprehension. It now says:

“In general, neurons harbor energetic/metabolic sensors such as AMP-activated protein kinase, which tends to restore ATP concentration by decreasing energy consumption while increasing energy production after different energetic challenges (Hardie, 2011, Hardie et al., 2012, Potter et al., 2010). This same mechanism has been linked to plasticity (Potter et al., 2015), where they were able to show that energetic stress can suppress LTP, while rescuing it by removing the energetic stress. This findings inspired our LTP energy-dependent plasticity rule”

**Comment 40**
* Why is everything exponentially dependent on ATP level? What is a reasonable level of 'sensitivity'? Looking at, for example, Fig 10, everything seems to be in a very small range of $A_t$.

**Answer**
Our model is phenomenological, which gives us the freedom to choose how to represent the phenomena. The exponential dependency on ATP levels is chosen to capture the non-linear effects of energy availability on synaptic plasticity.

**Comment 41**
* Some missing context (that makes the problem of links to data worse) and missing citations of previous literature. For example: Smith et al (Neuro Behav Rev, 2011) on human learning with metabolic differences, Padamsey et al (Neuron, 2022) on changing neuronal activity and behavior under metabolic restrictions, and Pache & van Rossum (Curr Opp Neurobiol, 2023) on learning rules that reduce energetic demands (also the paper from Li & van Rossum (Elife 2020) is missing). For more general context on the trade-offs between energy and neuronal/synaptic function in the context of optimality see e.g. Harris et al. (Curr Biol. 2015), Harris et al. (PLoS CB 2019), Mahajan and Nadkarni (eNeuro 2020), Jedlicka et al. (Open Biol 2022). Importantly, the authors should definitely mention papers specifically focused on synaptic plasticity and energetics from Jan Karbowski (J Neurophysiol 2019, J Comput Neurosci 2021) and also a previously published energy-related neuronal firing model from Fardet & Levina (Plos CB 2020).

**Answer**
We have improved the introduction based on reviewer's suggestions and other relevant research.

**Comment 42**
* Additive learning rules. There is some evidence that the skewed distributions of synaptic weights produced by multiplicative learning might be more energetically efficient (ie Goetz at al (PNAS, 2021), see also Roessler et al (Open Biol, 2023), Eggl et al (Comm Biol 2023). This might strongly impact the results given the interplay between energy consumption and learning. This is not meant as a major point but rather as a caveat that could be mentioned in the Discussion.

**Answer**
We appreciate the reviewer's observation. It is indeed an important consideration that multiplicative learning rules may lead to more energetically efficient synaptic weight distributions. While our current study focuses on additive learning rules, primarily because they allow us to mathematically formalize and understand the impact of energy-dependent plasticity rules at the network level, we acknowledge the potential benefits of multiplicative learning rules. Exploring the impact of energy constraints on these rules is part of our future work, and our model is designed to accommodate both additive and multiplicative modes.

**Comment 43**
* Figure 1 shows that reduced energetic availability increases the firing rate due to the inability of the cells to properly reset. In later results sections (ie page 20), it is assumed that reduced energetic availability reduces the firing rate (and not just through reduced LTP). How are these two phenomena to be reconciled?

**Answer**
Fig. 1 shows that reduced energetic availability, together with higher sensitivity to energy

imbalances ($\gamma$), increases the firing rate given the same input to the neuron. However, if the incoming current to the neuron decreases, that assertion is not necessarily true. Also, in Fig. 1, we assume only one neuron receiving external incoming current. In contrast, when we analyze the impact of metabolic constraints in the network in the reduced available energy scenario, there are two phenomena pulling in opposite direction: (i) the increasing firing rate of the neuron due to decreased available energy and (ii) the decrease in excitatory-excitatory weights through STDP, which globally decreases the excitatory activity (firing rate) in the network.

Possibly, the easiest way to understand what is happening, is to recognize that plasticity has a much stronger impact in the network than the neuronal sensitivity to energy imbalances ($\gamma$). Please note that synaptic plasticity sensitivity to energy imbalances directly defines the fixed point for the network (see Eqn. 41). The stronger impact of energy sensitivity at the synaptic and neuronal level can also be observed when comparing Fig. 9 (new Fig. 6). Keeping gamma constant and modifying eta (new Fig. 6) has a higher impact in the networks' dynamics, compared to keeping eta fixed and varying gamma (new Fig. 7)

**Comment 44**
* Much of the results are interpreted under the idea of 'balanced' ATP production and consumption, but the consequences of excess ATP accumulation are not really explored. It seems that it might lead to over-potentiation and instability, but it would be good to see this.

**Answer**
This is quite an interesting question. Literature is strongly biased to the study related to the lack of energy rather than the excess. In general terms, and beyond neural scope, excess of energy implies oxidative stress derived from mitochondrial metabolism (Pizzino et al., 2017).This stress can imply relevant damage to tissue if not properly counteracted by antioxidants. ATP concentration is related with the amount of glutamate release, being higher under ATP excess (Lanser et al., 2017). This means that when more ATP is available, more expenses will be induced due to glutamatergic activity. Also, microglia has been reported to be sensitive to ATP, modulating neuron's activity (Badimon et al., 2020). When more ATP is available, the less regulated the neurons are, leading to higher expenses. Entering into indirect evidence, metabolic adaptations of individual neurons strikingly resemble "homeostatic synaptic plasticity" or, more specifically, synaptic scaling (Barral et al., 2016; Penn et al., 2016) in which the activity of the network is increased or decreased after adding a blocker or enhancer (respectively) of synaptic activity for extended periods (48 hours) (Turrigiano, 2012; Turrigiano, 2008). In this context, the lack of activity (therefore increase in energy availability), is rapidly compensated by increasing synaptic weights increasing expenditure (Ibata et al., 2008).

In an *in vivo* approach, memory is known to be sensitive to energetic stress (Potter et al., 2010). Accordingly, food restriction in rats has demonstrated increased memory performance (Choi et al., 2021; Wiehler et al., 2022), while flies significantly increase sucrose intake after learning (Plaçais et al., 2017). An excess in energy availability induced by a high-caloric diet has shown the opposite effects (Treviño et al., 2015), showing that energetic stress could modulate behavior and cognitive abilities. In addition, non-hydrolysable glucose analogs such as 2DG in vitro promote LTP, an effect that is reverted by adding glucose (Zhang et al.,

2000). Other studies have shown that LTP is disrupted under low-energy conditions, such as glycolytic inhibitors or AMP mimetics (Potter et al., 2010).

**Comment 45**
* I don't understand what Fig 4 shows. Are synaptic connectivities or strengths distant dependent? This does not seem to be the case in the model.

**Answer**
No, synaptic connectivities are not distant-dependent. We think that Fig. 4 was not adding relevant information but rather distracting, thus we decided to take it out.

**Comment 46**
* Short-term presynaptic plasticity was not explored although the presynaptic energy/calcium/synaptic vesicle pool would affect the firing rates on the time scale that has been simulated in the paper (see e.g. Mahajan & Nadkarni, eNeuro 2020)

**Answer**
This is an interesting observation, and we agree that short-term presynaptic plasticity, which involves the presynaptic energy, calcium levels, and synaptic vesicle pools, could indeed affect the firing rates on the time scale simulated in our study. We will explore the impact of short-term plasticity in our model in future work.

**Comment 47**
* "experimentally, it is difficult to precisely measure ATP concentrations in single neurons or populations. To overcome this difficulty and to define a more general framework, here we will use percentage units to quantify available energy" That is fine but how was the value for the time constant tau_A determined? "K = 1/τA is the rate at which ATP can be produced in the neuron. Again, how is the model related to the data?

**Answer**
A detailed explanation of the EDLIF model with biologically realistic parameter values was given in Jaras et al., 2021. For a healthy neuron, the rate at which ATP is produced (K) is equal to 1 and was obtained while trying to keep our simplified EDLIF model close to the biologically detailed neuron model introduced in Le Masson et al., 2014. In the EDLIF article (Jaras et al., 2021), our model is compared against the leaky integrate-and-fire model using real data.

**Comment 48**
* The authors write: "Energy Homeostasis Principle (EHP) was introduced in [10, 11]. The rationale behind this principle posits that neurons, in meeting their metabolic needs, inadvertently address behavioral challenges as an epiphenomenon." Can the authors explain this sentence? Considering" behavioral challenges" as an epiphenomenon does not seem plausible. This should be made clearer or changed.

**Answer**
It is in fact challenging to explain it briefly. We understand the skepticism, especially when the traditional approach is a symbolic processing paradigm (as the evidence from fMRI proposes constantly with a strong modularims approach), and that most of research looks at leaving beings neglecting the fact that what is alive is an infinitesimal part of what has been

alive. In other words, that biological systems could be fairly developed randomly with some minor heritability restrictions, and we would still see smart-like machines which were developed without the direction of a smart mechanism. As such, when considering that living being mechanisms can be developed seemingly intelligently, but not necessarily, the assignment of functions to parts makes as much sense as assigning a function to a tree in a forest. The tree is simply there, its activity surely affects the forest, but is not intelligently designed to be there to fulfill a specific function.

The Energy Homeostasis Principle (EHP) stands from this naturalistic point of view. Considering that neurons are not there to fulfill a specific function, rather to deploy the set of actions determined by their structure restrictions. Under this scenario it is difficult to explain adaptive behavior, however it is still possible. For instance, Kagan et al., (2022) was the last article of several (Novellino et al., 2027, Tessadori et al., 2011, , Bruzzone et al., 2015) presenting how neural cultures can learn. This is a critical founding, as network architecture is lost during the culturing process. This means that there is a fundamental property in neurons which allows them to produce coherent behavior (i.e., avoiding obstacles, playing pong). The EHP predicts that this learning process is driven by metabolic constraints which modifies synaptic weights, and in turn network dynamics and ultimately behavior. For these specific experiments, indirect evidence supports this prediction (please see the EHP article for more details). In this context, EHP proposed that evolution has restricted neural architecture so that neurons will frequently converge to critical functions such as breathing, understanding that it is not always the case (e.g., sleep apnea, sudden death syndrome).

The EHP reinterprets previous evidence and proposes specific physiological mechanisms to be explored (such as synapsis energy dependence). Briefly, the energy Homeostasis Principle then suggests that when neurons solve their metabolic management (determined by their structure constraints or "neurons do what neurons do"), behavior is affected. This is rather straightforward, energy availability is known to affect synaptic weight, therefore network dynamics are affected, and then behavior is compromised. Understanding that we cannot extend too much this explanation and that both articles are available for reader to check, we have extended the sentence in a concise way:

"Attempting to establish the significance of energy management as a pivotal factor influencing neural systems, the Energy Homeostasis Principle (EHP) was introduced in~\cite{energy_principle, vicenco2022}. The rationale behind this principle posits that neurons, in meeting their metabolic needs, inadvertently address behavioral challenges as an epiphenomenon. EHP allows the reinterpretation of several physiological mechanisms. One of them is the synapsis, which is reinterpreted as an energetic control mechanism."

**Comment 49**
Minor:
* Reference 23 contains first, not last names.

**Answer**
The reference is now updated.
