# Supplementary material for: Unveiling the role of local metabolic constraints on the structure and activity of spiking neural networks
Source: PLoS Comput Biol. 2025 Jun 13;21(6):e1013148. doi: 10.1371/journal.pcbi.1013148 (PMC12201681; doi:10.1371/journal.pcbi.1013148)
Supplement: Attachment — Submitted filename: answers_to_reviewers.pdf [file pcbi.1013148.s011.pdf]

Comments to the Authors:
Please note here if the review is uploaded as an attachment.

Reviewer #1: NB: For a properly formatted version of this review, see the PDF attachment. All line numbers refer to the new manuscript, not the diff file.

Review PCOMPBIOL-D-23-01765 (R2)

Comment 1
This revised version of the manuscript is a significant improvement over the previous version.
Though I maintain that a proper analysis of the phase space and fixed points stability would have further improved the article, I think that the manuscript can be published close to its current state.
However, before the manuscript can be published, a few key issues, detailed below, must still be addressed.

Answer
We thank the reviewer. In the following pages, we answer all specific reviewer's comments.

Comments on the authors' replies
Comment 2
Comment 10

> Given that the reviewer has not given any concrete reason to perform such change, and that we disagree on the suggestion, we have not made the requested change. The aim of the studies is usually left by the end of the introduction, as we did it. We also think that the objective is properly described. Therefore we were not able to understand the rationale of the suggested change.

The aim usually comes before the summary of the content; here it seems to be split into two by the paragraph starting by "Through our mathematical analysis...", which feels strange to me. But this is indeed a matter of personal preference.

Answer
We appreciate the feedback. After consideration, we decided to retain the current structure as it aligns with our intended narrative flow. Thank you for sharing your perspective.

Comment 3
Distorted view of the field

> The discussion has been updated including references for the eLIF and mAdEx models, as well as an explanation of our decision for using the EDLIF model.

I appreciate the addition; however, my remark about the manuscript giving a distorted view of the field still stands.
In particular, there is another phenomenological model from [Chhabria2016] that is quite

similar (modeling ATP concentration) and has been used to model the influence of energy on network dynamics, so a more general comparison of the results to these models would also be relevant.
Finally, as the authors also cite conductance-based models from Le Masson and Jolivet, I am unsure why other works from e.g. Knowlton or Ullah, Wei and Schiff are not included, especially in the discussion about neurodegenerative diseases.

[Chhabria2016]: https://www.frontiersin.org/articles/10.3389/fneur.2016.00024/full

Answer
Thank you for highlighting these relevant articles. We agreed that our model shares similarities with Model 2 introduced in Chhabria 2016, particularly the adaptive voltage threshold given the ATP level, which can be shown to be mathematically equivalent to having an ATP-dependent reset potential when using the LIF model (Bugmann, 1997). However, we think the distinctions between EDLIF and eLIF (and mAdEX) described in comment 2 of the previous round also support our decision to use EDLIF instead of Model 2 in Chhabria's work. Specifically:

1. The presence of the sensitivity parameter γ in the EDLIF model allows us to investigate the impact of energy imbalances based on different neuronal sensitivities, enabling a comparison of how the same metabolic impairment can affect neurons differently. As far as we understand, this is not possible to achieve using Model 2 in his current formulation.
2. In Model 2, a spike induces an instantaneous reduction in available energy, thus the dynamic of spikes' energy consumption is reduced to an instant. We think this is an important distinction because energy consumption is not an instantaneous process and, in general, the time constants associated with neuronal energy consumption are much larger than the membrane time constants, and the rate at which energy is consumed impacts neurons' behavior. This is particularly relevant when studying neuronal metabolic production impairments, as the same total energy consumed per activity could lead to different behaviors in the neuron if the rate at which that energy is consumed differs.
3. Our purpose is to investigate the network's dynamics accounting for both action potentials and postsynaptic potentials on the neuron's energy dynamics (as shown in our Eq. 16). Under Model 2 current formulation, the energy dynamic doesn't account for synaptic transmission energy expenditure, thus making Model 2 unsuitable for our objectives. We acknowledge, however, that Model 2 could be modified to include synaptic transmission energy consumption.

We have updated our discussion to reference Chhabria's Model 2, as well as related works by Knowlton et al. (2018) and Wei et al. (2014), to further contextualize our approach.

C**omment 4**
Neuronal cultures

> a metabolic view of neural network activity is easier to assess in vitro using multielectrode arrays [where] it is reasonable to suppose that neurons present the possibility of a saturated network with an all-to-all connectivity.

Even for 500 neurons, **cultured networks do not display all-to-all connectivity**.
The typical connectivity is quite sparse, with around 100 connected neighbors per neuron [Soriano2008], [Barral2016].
The cited paper [39] refers to functional connectivity, not physical connectivity.
As I mentioned before, it is perfectly understandable for the authors to use an all-to-all connectivity to simplify the link between the mathematical analysis and the simulations, but they should not imply that this type of connectivity corresponds to standard *in vitro* structures.

[Soriano2008]: https://www.ncbi.nlm.nih.gov/pmc/articles/PMC2544527/
[Barral2016]: https://www.nature.com/articles/nn.4415

Answer
We thank the reviewer for this observation and agree that the manuscript could better clarify the distinction between structural and functional connectivity and our decision for an all-to-all structural connectivity.

When describing the network by an all-to-all connectivity, we are referring to its structural connectivity. To our knowledge, there are no structural connectivity estimations for MEAs. However, by visual inspection based on our experience working with neural cultures an all to all structural connectivity seems reasonable for cultures with 21 days or more (which is our future target). Understanding that we cannot back up this appreciation in previous literature, we just argue in favor of the decision based on the simplification of mathematical tractability. Importantly, despite the all to all structural connectivity, the network's functional connectivity was sampled with synaptic weights based on reported functional connectivity distributions for microelectrode array setups (Bettencourt et al., 2007), thus reflecting a biologically realistic connectivity pattern.

The manuscript has been updated to improve clarity.

Scientific remarks
Comment 5
Neglecting action potential energy consumption and energy relationship (Comment 22 and L 663)

The authors seem to have missed or forgotten one of the points of my comment 22 in the previous round, which was about the derivation of equations 41 and 42 by neglecting the action potential energy consumption.

I maintain that, given that the premise seems incorrect, these equations do not appear relevant and that the following statement, on line 663, is probably incorrect:

> both simulations satisfy the energy relationship defined by Eq. 42.

This equation relies on the hypothesis that $E_{ap}\overline{\nu} \ll E_{syn} \sum \overline{w} \overline{\nu}$.
The experimental reports we have for both values give $A_{syn} \approx 3 A_{ap}$, which

the authors have acknowledged in response to my initial comment.

In addition, the values given in figure 7 c and d are :

- $A_{ap} = E_{ap}\overline{\nu} \sim 4.5 \times 100 = 450$
- $A_{syn} = E_{syn} \sum \overline{w} \overline{\nu} \sim 0.5 \times 1700 = 850$

giving a ratio lower than 2, which does not satisfy the hypothesis leading to Eq. 42.

I don't think this is a major issue for the paper as a whole because this does not seem to be used anywhere (as far as I can tell), but if it is indeed incorrect, it should be removed.

Answer
Thank you very much for reminding us of this important point. The primary aim of Eqs. 41 and 42 is to illustrate the inverse relationship between excitatory-excitatory weights and excitatory firing rates, as this is a new relationship that emerges once energy constraints are included in the neuron and plasticity models. In our opinion, this inverse relationship is valuable to gain a deeper understanding and intuition on the relationship between energy constraints and neural networks dynamics. However, as you correctly pointed out, deriving Eqs. 41 and 42 from Eq. 40 by neglecting the action potential energy consumption is indeed incorrect, and we apologize for overlooking this correction in the previous round.

Using the $A_{syn} \approx 3 A_{ap}$ experimental relationship suggested on comment 22 of the previous round, and writing it in a more general form $A_{ap} \approx m A_{syn}$, we proposed an alternative way to state the relevant inverse relationship. This approach allows us to rewrite it as follows:

\frac{-A_{H} K ln(\alpha)}{\eta} \approx (m + 1) E_{syn} \sum_{syn. k} \overline{w}^k_t \overline{\nu}^k_t.
Thus,
\frac{-A_{H} K ln(\alpha)}{(m + 1) \eta E_{syn} } \approx E_{syn} \sum_{syn. k} \overline{w}^k_t \overline{\nu}^k_t

The updated equation shows the relevant inverse relationship between weights and firing rates. The manuscript has been updated accordingly.

As a side note regarding your estimation of A_{ap} and A_{syn} based on the information presented on Fig 7: The calculation of A_{syn} should include the synaptic energy consumption from both excitatory and inhibitory synapses. Based on your initial approximations, an updated estimation of A_{syn} would be as follows (with the firing rate given in kHz):
- $A_{syn} = E_{syn} \sum \overline{w}^{EE} \overline{\nu}^{E} + E_{syn} \sum \overline{w}^{EI} \overline{\nu}^{I} \sim 0.5 \times (1.7 + 0.55)= 1.125 %/s$.
Also, $E_ap = 4.1$, thus:
- $A_{ap} = E_{ap}\overline{\nu} \sim 4.1 \times 0.1 = 0.41 %/s$

giving a ration of, approximately, 2.7.

Comment 6
Oscillations (L 640)

The theoretical prediction for increased oscillations (point 3) feels rather hand-wavy though there are indeed reasons to expect increased oscillations. The explanations given from line 715 are much better in that regard and should be properly summarized in point 3 to replace the current phrasing. Ideally, the stability of the fixed point should have been analyzed to show if lower values of K lead to a phase transition from a stable to an unstable (saddle?) fixed point.
Figure 9 even suggests that the attractor dynamics may involve spiral fixed-points or more complex structures.

Answer
Following the reviewer's suggestion, we have revised and replaced the description in point 3 by a new explanation that summarizes the description starting from line 715.

Additionally, we analyzed the stability of the fixed point and included a new section in the supplementary information. Around the fixed point, the system is marginally stable. When K becomes sufficiently small, the eigenvalues turn into complex conjugates (see SI Fig 2), introducing oscillatory behavior into the system. This behavior aligns with the reviewer's suggestion of spiral-type fixed points. Regarding the appearance of saddle points, our analysis indicates that the fixed point does not transition into a saddle point as K decreases.

Comment 7
Comparison of η vs γ (L 798)

> In particular, the synaptic sensitivity (η) has a greater impact than the neuronal sensitivity (γ) on the network dynamics and fixed point.

I do not think that the current results are sufficient to back that claim.
In particular, though both variables appear in similar exponential terms, the range explored was not the same for both (η was set up to 100 while γ was only set up to 50).
At the very least, the authors need to explore the same range for both variables before making that claim.
Though we know that changes in γ do not change the position of the fixed point, they may change its stability.
To prove the initial claim, the authors would need to demonstrate that changes in η lead to several phase transitions or quantitative changes which cannot be achieved by changing γ, or that these changes would appear for unrealistic values of γ. As the analysis of phase-space and the fixed-point stability has not been performed, this claim cannot be made at the moment.

Answer
We agree that a broader exploration of γ values would strengthen our argument. Accordingly, we conducted additional simulations and have updated Fig. 7 to include a case with $\gamma = 100$ instead of the previous $\gamma = 50$ experiment.

Additionally, we have included a new section in the supplementary information where the stability of the system around the fixed point is analyzed. By examining Fig. 2 in the SI, it becomes clear that η has a more significant impact on the system's eigenvalues around the fixed point. Panels (b), (e), and (h) show that the eigenvalues change more significantly when varying η, compared to changes in the analogous γ variable (ϱ).

Comment 8
General remarks

Both K and $\tau_A = 1/K$ are used, which seems to provide unnecessary confusion. Unless there is a specific reason to keep both, please choose either one or the other throughout the text.

The use of the capital letter A to designate both energy (ATP) levels and energy consumption (A_{ap}, A_{syn}) is probably not the best choice. I would suggest using A only for the ATP level and replacing $A_{ap/syn}$ by another letter (e.g. c) and using $C(t) = \int c(t')dt'$ in equation 16.

It would be useful to adapt figures 6c, 7c, and 8c to test their overlap with the theoretical nullclines from figure 4.
This would enable us to see how well the theoretical predictions fit the simulation results.

Answer
In the original EDLIF publication, only K was used, without the need for τ_A. In this manuscript, however, we include τ_A to help readers to better understand and compare its impact with other time constants, such as τ_{ap} and τ^{A}_{syn}. Including both variables allows us to remain consistent with the original EDLIF publication while also providing greater intuition on how different time constants influence energy dynamics, as demonstrated in Eq. 16.

Additionally, we agree with the suggestion to replace $A_{syn/ap}$ for $C_{syn/ap}$, and the manuscript has been updated accordingly.

Following your recommendations, we have included the theoretical prediction described in Eq. 41 in Figs 6d, 7d and 8d. Although this is a slight adaptation of your suggestion, we believe that comparing the theoretical predictions in Eq. 41 with the results shown in panel d of Figs 6, 7 and 8 accomplishes the same goal with improved clarity. Thank you for proposing this change. It strengthens the connection between our theoretical and simulation results.
Specific remarks

Comment 9
L 166 : there is an ambiguity for the overline notation that usually means "average" (which is also used later multiple times with that meaning, e.g. for $\overline{\nu}$), I would suggest to use another notation, e.g. $\tilde{w}$.

Answer
The text has been updated following the suggestions to correct the ambiguity.

Comment 10
L 177 and L 187 : replace "For finding" by "To find".

Answer
The text has been updated.

Comment 11
Eq 14 : replace "spike s" by "spike f" to be consistent with eq. 16?

Answer
The text has been updated following the suggestion.

Comment 12
Eq 16 :

- the terms are the integrals of the action potential and the synaptic consumption rates, not the rates themselves, so A_{ap} and A_{syn} should not be used together with the braces; use something like "action potential contribution" and "synaptic contribution" instead, or define C_{ap} and C_{syn} and use these.
- remove the Heaviside function from the action potential contribution, as it should have been integrated away.

Answer
We have updated Eq. 16 to improve clarity by replacing A_{ap} and A_{syn} with C_{ap} and C_{syn}, and we have also included the word “contribution" in the equation. Additionally, we have removed the Heaviside function from the action potential contribution.

Comment 13
L 218 :

> This same mechanism has been linked to plasticity [29], where they were able to show that energetic stress can suppress LTP, while rescuing it by removing the energetic stress.

The end of the sentence is not clear to me, from article [29] I believe the sentence should read "while treatments targeting energetic pathways can rescue it by removing the energetic stress."

Answer
The text has been updated following the suggestions.

Comment 14
L 270 : the last value should be n_I and not n_{in}.

Answer
The text has been corrected.

Results :

Comment 15
* Default values for parameters γ, η, K should be provided in a 2nd table in the supplementary material so we know which values are used when the other parameters are changed.
At the moment, they are mostly specified in the main text or the figure caption, but some values are missing for specific simulations (notably K).

Answer
We have included a second table in the supplementary material, which provides the default values for the parameters γ, η, and K.

Comment 16
* L 517 :
- n_{ex} should be n_E
- why is ν_{max} given as 0.125 and not 125, since the unit is Hz?
- A_c is an energy consumption rate so its unit should be homogeneous to $\%/s$

Answer
Thank you for finding this error. The units for A_s and A_c should indeed both be [%/ms] (please see Eqs. 6 and 7. Also, K units are 1/ms). Consequently, the firing rate should be measured in KHz when calculating A_c, explaining why we use 0.125 instead of 125. Where possible, we have opted for Hz units, as we believe these are generally more familiar to readers than KHz.

The manuscript has been updated accordingly.

Comment 17
* Figures 6, 7, 8, please add a label stating that the colorbar give the average ATP level in the steady-state (or explain what it is if it is something else). I would also suggest to not fill the symbols in the legend for panels c. For figure 8, please rescale the colorbar to make the differences visible (if it is indeed the average value, I imagine it should not go below 96).

Answer
Figs. 6, 7 and 8 have been updated following the suggestions.

Discussion :
Comment 18
L 855 :

> neurodegeneration has been treated by increasing mitochondrial energy production or affecting activity through neurotransmission (e.g., pregabalin to inhibit or levodopa to increase activity)

The use of levodopa has more complex results than simply to "increase activity": in the basal ganglia, dopamine either excites or inhibits inhibitory neurons that will themselves inhibit the activity of other neurons.

Answer

We thank the reviewer for the precision. Indeed, the impact of drugs on network dynamics is more complex than on a single neuron. Nonetheless, our arguments prevail. Regardless of which specific neurons increase or reduce their activity, it is the activity what is modulated.

We now have updated the text to “(e.g., pregabalin or levodopa to modulate activity)”.

Comment 19
L 860 :

> general terms,as currently done

missing whitespace

Answer
The manuscript has been corrected.

Comment 20
L 862 :

> Neurons are highly oxidative and selectively low-tolerant to oxidative stress [44].

I don't think this sentence is correct in English and would suggest rephrasing to something like "and some neuronal populations can be vulnerable to oxidative stress".

Answer
Thank you very much for the suggestion. We have updated the text accordingly.

Comment 21
L 863 :

> They maintain a constant expenditure regardless of activity level [45].

I don't think this corresponds to the current consensus on neuronal metabolism, see e.g. [Watts2018].
Furthermore, [45] states that the NKP may drive mitochondrial activity, not that the energy expenditure is constant.

[Watts2018]:
https://www.frontiersin.org/journals/molecular-neuroscience/articles/10.3389/fnmol.2018.00216/full

Answer
We thank the reviewer for pointing this out. The Watts 2018 evidence was obtained in vivo, under conditions which are fairly different regarding the access to oxygen and dynamics related to the neurovascular coupling. Despite the relevance of such literature, we already framed our model under an in vitro paradigm, such as the one reported by Baeza-Lehner. et al (2019).

However, the reviewer is right, we must correct the sentence. What is constant are ATP levels. Therefore, availability seems fairly stable regardless of the activity. We apologize for the mistake, we have now corrected the draft. Also, it is true that the stability of ATP can be discussed. There is abundant evidence presenting that ATP is not stable, however, to our knowledge this evidence used non-physiological stimulation. Therefore we have now edited the drafts to state “To our knowledge, they maintain a constant availability regardless of activity level [45] when using physiological-like stimulation.”

Comment 22
L 923 :

> the ratio between total energy consumed by action potential and synapses is 60 : 20

This sentence is misleading as it seems like 60 corresponds to the contribution of the action potential. Please rephrase it into something like "the ratio between the total energy consumed by synaptic transmission and the energy consumed by action potential generation is $C_{syn} / C_{ap} = 3$."

Answer
The sentence has been corrected to improve clarity.

Comment 23
L 926 :

> In general, previous neuronal and synaptic models do not account for energy dependencies (some exceptions can be found in [21, 49, 53]).

while it is true that most publications in computational neuroscience do not consider energetic constraints, the part in parenthesis again provides a distorted image of the field. There are phenomenological models on the subject dating back to 2016 and, if one also considers conductance-based models (which the authors apparently do given that they cite [21] and [53]), there are many more papers specifically dedicated to the subject, notably from the authors mentioned in my first comment, some of them discussing a phase-space analysis of their models [Knowlton2018], [Yao2018].

[Knowlton2018]: http://www.physiology.org/doi/10.1152/jn.00351.2017
[Yao2018]: http://aip.scitation.org/doi/10.1063/1.5018707

Answer
We have updated the text to provide further references to previous neuronal models that account for energy dependencies.

Code
Comment 24
this is unrelated to the current paper, but, since I was now able to test the code, I noticed that it uses weighted clustering coefficients. I suggest having a look at these articles if there are plans to use weighted clustering in future work, to make sure that the definition chosen is indeed appropriate given the scientific question:

-
[10.1103/PhysRevResearch.3.043124](https://journals.aps.org/prresearch/abstract/10.1103/PhysRevResearch.3.043124)
- https://hal.science/hal-04427288

Answer
Thank you very much for this suggestion and piece of information.

Reviewer #2: Review:
Upon review of the updated article, I still have many concerns.

Comment 25
Response to Comment 30.
I'm afraid this is not accurate. See, for example, Akil et al. 2021 PloS Comp Bio, in which they study STDP in balanced spiking networks. "E/I balanced networks" has a particular connotation in terms of the parametric regime regarding synaptic weights, which may be assumed fixed, or plastic. Since this is not what this paper is addressing (clearly, from Fig. 5, in which the 'non-metabolic' situation explodes except for refractory effects), lines 263/264 (and other instances) should be modified to omit the term "balanced".
A more general related concern is what the role of inhibition in this model is, at all. From Fig 5, the net inhibitory current is orders of magnitude below that of excitation, suggesting that inhibition is really having little to no effect on the excitatory dynamics. This then begs the question, as to whether the 'metabolic' effects are justifiably the main actors, and what, aside from the silencing of some neurons in Fig 6, is the role of inhibition here? I believe that in reality, they are not doing much, and some support for this is the explanatory figure attached following Comment 31 in the response, where an excitatory-only network was simulated. In general, how robust is this, if inhibition is removed? It may be justified to include a simulated excitatory-only network in the text for comparison.

Answer
To avoid any confusion on this topic, we have clarified most references to "E-I balanced networks" in the previous revision, modifying them to "E-I networks." However, one instance of "E-I balanced networks" remains on line 265, where we are not describing our specific network but rather discussing relevant biological observations. The text states: "There is biological evidence suggesting the existence of excitatory-inhibitory (E-I) balanced networks in different parts of the brain, such as the CA3 region of the hippocampus, basal ganglia, and the primary visual cortex~\cite{rockel1980, hendry1984, parnavelas1991, contreras2004}. Thus, E-I networks appear as a suitable model to explore neural networks' dynamics and structure." To the best of our knowledge, this claim is accurate. We have carefully reviewed the manuscript for additional references to "E-I balanced networks" and believe none remain. If any were overlooked, we apologize and would be grateful to the reviewer for pointing them out so we can make the necessary corrections.

Regarding the significance of inhibitory neurons in our network, their role in the energy dynamics, and consequently in shaping network architecture, is both interesting and crucial. Since inhibitory neurons contribute to energy consumption in postsynaptic neurons, excitatory neurons that receive more inhibitory postsynaptic potentials (IPSPs) are less able

to form strong synapses with excitatory presynaptic partners compared to those receiving fewer IPSPs. This phenomenon arises from the incorporation of energy constraints at both the neuron and synapse levels, as described in Eqs. 41 and 42. Notably, these equations illustrate that the presence of inhibitory neurons influences not only the network dynamics but also its architecture.

The following rationale highlights the relevance of inhibitory neurons in the network. Imagine a scenario in which excitatory-inhibitory weights or the firing rates of inhibitory neurons are increased such that the excitatory-inhibitory term on the right-hand side of Eq. 41 (second term) approaches, but does not exceed, the left-hand side. This increase in inhibitory activity would result in a reduction in the firing rates of excitatory neurons. To ensure that excitatory neurons remain active during this experiment, an external current could be applied to keep their firing rate above zero. Under these conditions, energy constraints would drive excitatory-excitatory weights close to zero, thus enforcing a network with minimal excitatory-excitatory synapses to satisfy Eq. 41.

This example illustrates the importance of inhibitory neurons in structuring the network. Including inhibitory neurons not only results in a more biologically realistic model but also provides a general framework that can extend to other types of plasticity, such as inhibitory-inhibitory, excitatory-inhibitory, and inhibitory-excitatory interactions. We believe this framework offers a valuable contribution, deepening our understanding of how energy constraints affect spiking neural networks.

Comment 26
Response to Comment 31.
I apologize that my point was not well made, given the author's response. To clarify, I was suggesting that a simplified model could do just as good as a homogeneous one in some cases, with the benefit of explaining some of the more complex oscillatory behavior or the like. Just a thought.
Importantly, a significant omission in the main text are any plots showing the evolution of the E-E weights, which were shown in the rebuttal in an explanatory fashion. I think weight evolution plots should be included in the main text, and amended to better illustrate the claims in the main text. This is especially true considering that the weights appear in only one case (\eta = 0) to have actually stabilized.

Answer
We appreciate the reviewer's suggestion to explore a simplified model and will consider it in future developments of our theory.

Regarding the evolution of weights, the decision not to plot them is primarily due to one key reason. As detailed in our mathematical analysis, the focus of our work is on the fixed point of the network. We believe it is more valuable to present plots that align with and can be directly compared to our theoretical framework. This approach enables us to explain the observed behaviors in the plots and provide a precise mathematical correlate to support these observations.

It is important to highlight that the weights converge in almost all experiments, with the sole exception being the acutely impaired case (\$K=0.1\$). In all other cases, the average firing

rate (panel b in Figs. 6, 7, and 8) converges and then remains stable. This stability in the firing rate is also true for the energy level. The stabilization of both the firing rate and the energy level directly results from the weights reaching equilibrium, where the average Δw equals zero. The convergence and stability of the weights is a crucial condition for the stability of both the firing rate and the available energy in the network. This requirement is explicitly addressed during the derivation of the network’s fixed point in lines 437-44 (“Eq. 37 holds if the neuron is [..]”). Therefore, demonstrating that the network reaches the fixed point for the firing rates and available energy serves as evidence of weight stability. Nevertheless, to provide deeper insight into the system’s dynamics near the fixed point, we have included a new section in the supplementary information that analyze the fixed point’s stability.

Comment 27

Response to Comment 36.

The Heaviside function is still present from Eq 10 on, even though the integral of \epsilon from 0 to \infty takes care of this. Alternatively, if the constraint on the epsilons is relaxed to -infty to infty then the Heaviside is fine.

Answer

We appreciate the opportunity to clarify our rationale for including the Heaviside function in Eqs. 10–15.

When evaluating Eq. 10 for $t’ < t_s$, without the Heaviside function, and using an exponential kernel $\epsilon(t) = e^{-t/\tau}$. the synaptic contribution becomes:

$C_{syn}(t’) = E_{syn} \tilde{w} e^{-(t’ - t_s)/\tau}$

Since $t’ - t_s < 0$, this implies $C_{syn}(t’) > E_{syn} \tilde{w}$. Without the Heaviside function, $C_{syn}(t’)$ could grow arbitrarily large, which is inconsistent with the model’s assumptions. By including the Heaviside function, we prevent this situation and ensure the model behaves as intended. That is the reason why we include the Heaviside term in Eqs. 10-15.

However, we acknowledge that Reviewer 1 correctly pointed out the presence of an extra Heaviside term in Eq. 16. (see comment 12 in this letter). We have removed the term in Eq. 16.

We hope the previous explanation clarifies our reasoning.

Further concerns:

Comment 28

1. It would be nice to have some discussion of the shape of the rate to A curve (eq. 5), which shows that more ATP leads to reduced firing (e.g. from Jaras et al 2021). Also, some discussion of the locality of this solution, since in the A \to 0 limit there would still be a positive firing rate. Further, recent work from Chintaluri & Vogels (2023) suggests that increased ATP should lead to spike discharge, which would imply the opposite trend of this curve.

Answer

Thank you for this valuable comment. Indeed, a deeper discussion of the EDLIF model can be found in Jaras et al., 2021. In particular, Fig. 4 in that article illustrates the relationship

between A and the firing rate. However, in this article, we focus on energy-dependent synaptic plasticity and the dynamics of spiking neural networks under metabolic constraints.

Regarding the $A \to 0$ limit, the typical approach is to prevent the neuron from spiking if its energy falls below a certain threshold or to define the neuron as "dead" if the conditions for an action potential are met but $A=0$ (e.g., see Fardet et al., 2020). Since we did not simulate or explore the neuron model in this regime, we do not discuss this limit in the article.

Thank you for bringing up the connection to the work by Chintaluri & Vogels. They focus on describing spontaneous firing rates as a mechanism to increase energy consumption and prevent high ROS levels, which can damage the cell. This view on spontaneous firing rates in neurons is fascinating and consistent with the Energy Homeostasis Principle, our theoretical frame. In our framework, if the energy level exceeds the energy equilibrium $\breve{A}$, synaptic weights and firing rates tend to increase, thus increasing energy consumption.

Another interesting phenomenon related to energy and neuronal dynamics is how low ATP levels reduce the rate at which sodium and potassium concentrations are restored, leading to sodium accumulation inside the cell. This sodium accumulation keeps the neuron depolarized, making it more easily excitable. This is the phenomenon we emphasize while modeling the EDLIF neuron, and several studies in the literature align with these behaviors (e.g., Le Masson et al., 2014, Chhabria et al., 2016, Fardet et al., 2020). While we acknowledge the reviewer's suggestion to enhance the model by incorporating additional biological complexities, we want to highlight that the primary focus of this article is on energy-dependent plasticity and the dynamics of networks under energy constraints. The EDLIF model is well-suited for this purpose, and we explain our decision in the "Limitations & Future Directions" subsection of the discussion.

Comment 29
2. Given your definitions for w and v, eq. 24 is not correct, since the dimensions are mismatched. Should be either v*w or w should be defined as [w_ee, w_ei; w_ie, w_ii].

Answer
Thanks for pointing this out. We acknowledge the error in the description of the dimensions for w^{IE} and w^{EI}. To clarify, w^{XY} represents the matrix of synaptic weights for connections from population Y to population X, with dimension $n_X \times n_Y$. Therefore, w^{IE} has dimensions $n_I \times n_E$, while w^{EI} has dimensions $n_E \times n_I$.

We have corrected the text accordingly.

Comment 30
3. Lines 323/324 (after eq 27). You state that one needs constant weights for constant firing rates. This isn't correct - you've concluded you need constant weights in expectation.

Answer

Thank you for your suggestion to enhance the precision of our wording. We have updated the text to reflect the reviewer's recommendation.

Comment 31
4. Line 483 and on… could be illustrated in Figure 4 better to help the reader. Is Fig. 4 the actual solution to an instantiation of the equations or just an illustration? Line 530 and the caption suggest it's an illustration. If it's not, the parameters which were used to compute it should be provided.

Answer
As stated in the text (line 488), Fig. 4 is intended as a conceptual illustration of the intersection between the inverse relationship between excitatory-excitatory weights and excitatory firing rates (Eq. 42) and the direct relationship described by the neuron's activation function (Eq. 25). Starting from line 478, the primary description emphasizes these direct and inverse relationships, which Fig. 4 is designed to illustrate. Specifically, the red curve represents a classical inverse relationship between two variables, while the gray curve shows the direct relationship between E-E weights and excitatory firing rates.

All relevant information detailed from line 483 onward is summarized in the caption for Fig. 4. In our view, the figure includes all essential information to understand our framework and the role of the critical variables (such us K, γ, η) in determining fixed points in spiking neural networks under metabolic constraints. This figure was updated in a previous revision, and we believe the current version is effective. We appreciate the reviewer's suggestion; however, in the absence of specific feedback on what is unclear or need further explanation is provided, we prefer to retain the figure as it is.

Comment 32
5. A key point the authors emphasize is the silent neural population that emerges. However, no robustness arguments are explored here. How robust is this to changes in the initial conditions? Mean of the exponential distribution? Or, different distributions altogether e.g. gaussians with different means and variances? In a related vein:
-Line 553 and Fig 6. These oscillations appear to sample the firing rate floor. Are more and more neurons falling silent each time until enough are dead that the remainder stabilize at some equilibrium? Line 574 is also incomplete. Why is their energy consumption not zero? Because of incoming activity? Please clarify.
-Line 581. "Silent neurons emerge as a consequence of energy constraints". Is this true in general? How sensitive is this to the initial weight distribution? If the initial E-E weights are weaker (or, the I to E weights stronger) is this still the case?

Answer
We recognize the importance of this observation and are happy to clarify. Silent neurons still consume energy due to synaptic transmission, which is driven by the action potentials of neighboring neurons.

The primary contribution of our work lies in introducing the energy-dependent STDP rule and identifying the fixed point of the system. While we also simulate spiking neural networks to validate our theoretical findings, conducting exhaustive simulations to test all parameter configurations falls outside the scope of this study.

Our aim is to establish a general framework to explore the effects of various neuronal and synaptic energy constraints at the network level. Although a detailed exploration of scenarios leading to the emergence of silent neurons is on our agenda for future work, a comprehensive analysis of all such cases is beyond the goals of this manuscript.

To provide deeper insights into the impact of metabolic constraints on network dynamics, we have added a stability analysis section in the supplementary information. We hope this addition addresses some of the reviewer's concerns and enhances the clarity of our findings.

Comment 33
6. Fig 6. It appears that the blue (\eta = 30) population is also separating to some extent, especially in the E-E weights. If the simulation was run longer would they continue to diverge?

Answer
This is an interesting question. The populations do not diverge if the simulation runs for a longer period. To demonstrate this, we extended the simulation duration to more than five times the original period (100000 ms instead of 15000 ms), and the results remained stable, as shown in the attached Figure.

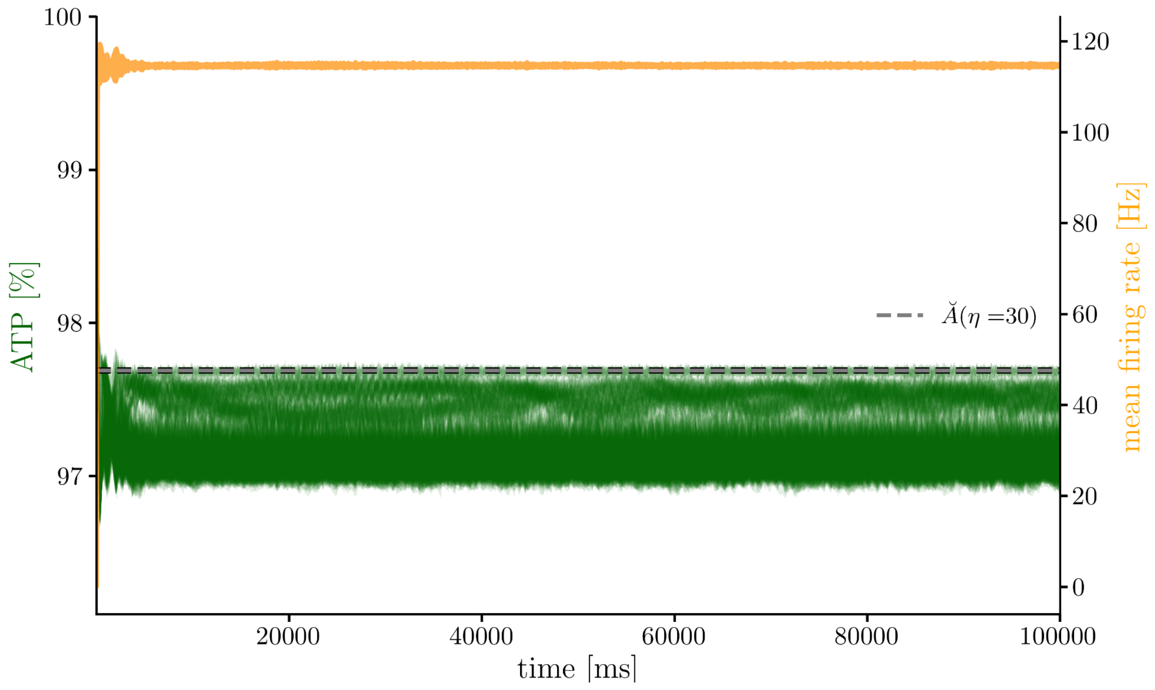

Furthermore, with the stability analysis included in the supplementary information, once the network reaches the fixed point, the state of the network should remain close to it. Therefore, the divergence of the two populations after reaching equilibrium goes against this expected behavior.

Comment 34
7. Figure 7d and similar (e.g. Fig 8d): plot the theoretical constraint line(s) from eq. 42 to demonstrate the accuracy of the result. Otherwise, the statement in line 613 is not so clear, just that they covary.

Answer
Thank you for this suggestion. The theoretical constraints from Eq. 41 are now included in panel (d) of Figs. 6, 7 and 8. Additionally, we have refined Eq. 41 to incorporate reviewers 1's suggestions. We believe this update enhances clarity and facilitates comparison between the theoretical predictions and simulation results

Comment 35
8. Figs 7/8. It is virtually impossible to see the color of the points on the current plots. If this is important to show together with the net currents, please find another way to plot the effect. And, please label the color bar on the plot.

Answer
We have adjusted the marker sizes to improve visibility of the color within each marker, making it easier to distinguish. Additionally, we have added a label to the color bar for clarity.

Additional points:
Comment 36
1. A_B is used for both basal consumption and basal production. Unless these are always the same (in which case it should be justified) these should be denoted differently.

Answer
The text has been updated following the reviewer's suggestion. Eq. 8 now uses C_B in place of A_B to describe basal energy consumption.

Comment 37
2. Line 373 the upper bound should be exp(-\eta) not 1.

Answer
Following Eq. 20 under additive plasticity rules (\mu_{\pm} = 0}, and with initial condition $A(t=0) =A_H$ as stated in the manuscript, we obtain the following results as $Delta t \to zero$:
peak amplitude for Δw potentiation = λ
peak amplitude for Δw depression= $\lambda \alpha$

Consequently, if $\alpha \in [0, 1)$, then peak depression is weaker than peak potentiation. Equivalently, peak potentiation is stronger than peak depression when $\alpha \in [0, 1)$, as described in the text.

We believe the text is accurate and that our formulation is correct. However, if there is something we may have overlooked, please provide specific details regarding the potential error or reasoning for an alternative upper bound so we can ensure the manuscript's accuracy.

Comment 38
3. Line 519. But what, then, is a reasonable E_{syn}?

Answer
As cited in our draft, the study by Howarth et al., 2012 supports a $20/60$ ratio for C_{ap}/C_{syn}. This ratio serves as a basis for estimating a reasonable value for E_{syn}.

We can estimate the average energy consumption in the neuron for action potentials (C_{ap}) and postsynaptic potentials (C_{syn}) as follows:
- $C_{ap} \approx \nu E_{ap}$
- $C_{syn} \approx \nu E_{syn} N <tilde{w}>$

where \nu is the average firing rate, N the number of neurons in the network, and $<\tilde{w}>$ the average value of the absolute and normalized synaptic strength ($w\w_{max}$, as defined in the manuscript). In our simulations, we initialize the weights following an exponential distribution with scale parameters $\beta = 5$ and $w_max = 100$, so $<w\w_{max}> = 1/20$.

Then, the ratio between C_{ap} and C_{syn} is:
$\frac{C_{ap}}{C_{syn}} = \frac{\nu E_{ap}}{N \nu E_{syn} <\tilde{w}>}$

If we force the $C_{ap}/{C_{syn}$ ratio to be equal to $20/60$ and match the results shown in Howarth et al., 2018, we arrive to the following relation:

$E_{ap} = \frac{N <\tilde{w}>}{3} E_{syn}$,

where $(N <\tilde{w}>)/3 \approx 8.3$ (500/60 given the parameters in our simulations).

Because we are interested in simulating our networks with variables more representative of biology, after the first round of reviews we decided to run all the simulation again using $E_{ap} = 8.2 E_{syn}$, thus aligning our numerical experiment with the energy consumption ratio presented in Howarth et al., 2012.

We hope this detailed explanation clarifies our decision regarding the E_{syn} values. Additionally, we have updated the text to explicitly reference the biological C_{ap}/C_{syn} ratio, specifically in Eqs. 41 and 42 and the preceding text.

Comment 39
4. Line 670. I don’t find this very clear to “contrast”; neither scrolling back and forth, nor the clear differences in the axis scales. If this is important, please find a clearer way to show it with a plot.

Answer
We appreciate the suggestion. In Fig 7 c ($\gamma = 20$, purple curve), excitatory firing rates range from approximately 90 to 110 Hz. Conversely, in Fig 8 c ($K = 0.7$, blue curve), excitatory firing rates vary from approximately 50 to 90 Hz. To clarify the comparison of firing rates in panel c of Figs. 7 and 8, we have included a reference to the respective curve colors and firing rate ranges in each figure. We hope this modification enhances clarity.

Comment 40

5. Line 383 this ‘potentiation-depression loop’ point is not well-illustrated in Fig 2, as suggested by the parenthetical. The figure could be updated to make this clearer.

Answer
Fig. 2 has been updated to provide more clarity regarding the potentiation-depression loop. Specifically, we now illustrate how the weight drift varies for different η values and postsynaptic energy levels, driving the postsynaptic energy level towards the equilibrium value $\breve{A}$.

Comment 41
6. I find the second paragraph of the introduction (lines 12 - 25) does not add anything of interest. I don’t think anyone believes that energy management is not important for normal brain function. Neuronal activity drives behavior, and requires energy to do so. So fMRI is correlated with energetics by definition.

Answer
We appreciate the reviewer’s comment and understand their perspective. However, we believe this paragraph serves an important role in setting the broader context for our work. While the relevance of energy management for neural function may seem self-evident, this paragraph provides a brief review of key literature and emphasizes the interplay between energy dynamics and behavior, which underpins the need for models like ours. For these reasons, we have opted to retain it.

Comment 42
7. Section beginning on Line 497. Fig 5 panels b,c are not discussed. Given that they show up later, they should be introduced better. Please label the color bar. How sensitive are these results to the choice of time averaging at the end of the simulation?

Answer
Thank you for pointing this out. We agree that Fig. 5 panels (b) and (c) require better integration. We have updated the text to introduce these panels more clearly, emphasizing their purpose for comparison and completeness. Additionally, the color bar has been labeled.

Regarding the sensitivity of the results to the choice of time averaging: for panels (b) and (c) in Fig. 5, as well as panels (c) and (d) in Figs. 6–8, we selected an averaging window of 1500 ms (10% of the total simulation duration) to ensure robustness. This window is significantly longer than the neuron's membrane time constant (20 ms), allowing us to capture stable dynamics effectively. Generally, results at the end of the simulation are stable, so the calculated firing rates remain consistent with variations in the averaging window.

However, it is worth noting that if the averaging window is too small, minor variations in its length can lead to noticeable changes in firing rate calculations. Similarly, if the network has not yet reached a fixed point, the averaging window’s duration will have a greater influence on the results.

Comment 43
8. Line 819. I don’t think this is a compelling argument for why certain neurons fire so sparsely (or, here, not at all). Indeed, in the brain there is also ample inhibitory plasticity

which could jointly adjust various set points. Further, despite it being a common assumption in models, a sustained 100Hz firing rate fixed point is not the goal of neural circuits. It also wouldn't explain why those neurons exist in the first place, and from a neural coding perspective leaves a lot to explain.

Answer
We understand that metabolic constraints alone may not fully explain sparse firing in certain neurons. Of course, other reasons may be taking place in vivo or in vitro, as other mechanisms could be at play in both in vivo and in vitro settings. However, we believe our model provides a plausible explanation for sparse activity, grounded in the interplay between energy constraints and network dynamics.

While inhibitory neurons indeed play a role in moderating network activity, they also increase energy consumption in their postsynaptic partners. Thus, if substantial inhibitory activity is necessary to balance high excitatory firing, it is possible that neurons could not sustain the resulting energy demands.

In our framework, when metabolic constraints are applied, some neurons naturally reduce their firing rates, contributing to network stability. Our reasoning is that, assuming redundancy in the brain, if all neurons were constantly active, their combined energy demands would likely be unsustainable. Under our model, certain neurons remain silent in order to facilitate an energy equilibrium among active neurons. Therefore, we hypothesize that this silent behavior in certain neurons emerges as a local adaptation to energy constraints, enabling those remaining active to maintain energy balance.

We believe this hypothesis is well-supported by our mathematical framework and simulation results. As such, we have retained this hypothesis in the manuscript, presenting it as one plausible explanation for sparse neuronal activity.

Reviewer #3:
Comment 44
The authors have generally responded well to my comments and I believe that the paper would be suitable for publication if the other reviewers believe that their comments have been addressed. The new manuscript makes the assumptions behind the model much clearer in the cases where data is limited or unavailable.

Answer
Thank you for your positive feedback and for recognizing the improvements in our manuscript. We are glad to hear that our revisions have clarified the assumptions behind the model, especially in cases where data is limited or unavailable.

We greatly appreciate your constructive feedback throughout the process.
