# Supplementary material for: Unveiling the role of local metabolic constraints on the structure and activity of spiking neural networks
Source: PLoS Comput Biol. 2025 Jun 13;21(6):e1013148. doi: 10.1371/journal.pcbi.1013148 (PMC12201681; doi:10.1371/journal.pcbi.1013148)
Supplement: Attachment — Submitted filename: answers_to_reviewrs.pdf [file pcbi.1013148.s013.pdf]

Reviewer #1: NB: For a properly formatted version of this review, see the PDF attachment.

Review PCOMPBIOL-D-23-01765_R2

Comment 1
This new revision further improved the quality of the article and answered my previous reservations.
I think this work will prove to be a valuable contribution to the field of neuronal metabolism.
Apart from minor typographic issues (that can be addressed without the need for another review), I consider that the article can be published as is.

Answer
Thank you very much for your thoughtful review and kind words. We truly appreciate your time and effort in providing valuable feedback, which has helped us refine our work.

Minor remark
Comment 2
> To our knowledge, they maintain a constant availability regardless of activity level [45] when using physiological-like stimulation.

"physiological-like" was not clear to me before I read the reply to my comments.
Maybe something like "when applying stimulations that do not exceed physiological levels" would be clearer?

Answer
Thank you for the suggestion. We have reworded the sentence for clarity: *"when applying stimulations that do not exceed physiological levels".*

Typographic remarks
Comment 3
The firing rate vector $\boldsymbol{\overline{\nu}}_t$ (Eq. 24 to 27 and in Eq. 38) should be made **bold** using ``\boldsymbol`` to show that it is a vector, according to the authors' typographic choice.

AnswerWe have adjusted the firing rate vector $\boldsymbol{\overline{\nu}}_t$ to bold using ``\boldsymbol``.

Comment 4
Lines 339-340 should read: "the network architecture is define**d**"

AnswerThe sentence has been corrected to read: *"the network architecture is defined"*

Comment 5
Line 858, it should be "levodopa" and not "levopoda".

AnswerWe have fixed the typo, changing "levopoda" to "levodopa".

Reviewer #2:
Comment 6
Response to Comment 25.
I'm not convinced. Fig 5 shows that in the 'base' case, inhibitory inputs are two orders of magnitude below excitation, but the responses saturate due to refractory period alone, and the energetics stabilize. Hence it seems that the energy constraints are doing the work of stabilizing the system. In principle there is nothing "special" about the inhibitory term in eq. 41; so if one were to split a purely excitatory population in two with one subset having plastic and the other non-plastic weights, you could play the same game as in eq's 40 & 41 and separate the non-plastic and plastic subsets. Further, I understand the thought experiment in the rebuttal, but it seems deeply contrived. For example, if one considers assemblies of neurons in cortex, E and I neurons may strongly co-cluster. By the same logic, assuming strong feedforward drive to both E and I, I neurons would cause the strong assembly connections to weaken. If one believes in, e.g. assemblies as storing memories, this would seem to be a problem.

Answer
Figure 5 shows that in a network without energy dependencies, inhibitory inputs are two orders of magnitude lower than excitatory inputs **after excitatory neurons saturate**. However, at the beginning of the simulation, excitatory and inhibitory synapses are sampled from the same distribution, meaning this difference emerges over time.

We acknowledge the reviewer's point that, in principle, a purely excitatory population could be split into two groups—one with plastic and one with static connections—similar to the separation shown in Equations 40 and 41. However, we argue that inhibitory neurons and synapses play a crucial role in network stability from a metabolic perspective. They provide an additional mechanism to help the network recover from energy challenges. Within our framework, when energy is scarce, both excitatory and inhibitory neurons become more sensitive. For excitatory neurons, this results in a positive feedback loop—stronger excitation in response to lower energy—which can drive the network toward even greater excitation, potentially exacerbating the energy deficit. In contrast, inhibitory neurons introduce a negative feedback mechanism that suppresses network activity, with this effect intensifying as energy levels decrease. This dynamic suggests that inhibitory neurons offer an essential regulatory mechanism for energy homeostasis.

Regarding memory, we acknowledge that neural memory mechanisms remain largely elusive. The reviewer's comment draws a parallel with *catastrophic forgetting* in artificial neural networks (ANNs) (French, 1999), where learned information is lost due to changes in network structure when new information is acquired. This is a fundamental challenge in connectionist models, and various strategies have been proposed to mitigate it (Kirkpatrick et al., 2017). However, the biological mechanisms that prevent catastrophic forgetting are not yet fully understood. What we do know is that both cortical and subcortical structures, such as the hippocampus and amygdala, play essential roles in memory formation and consolidation (Sridhar et al., 2023). Current neuroscience research suggests that cellular mechanisms, often not included in computational models, may be crucial in preventing catastrophic forgetting (Sridhar et al., 2023). Furthermore, memory consolidation involves coordinated long-range activity between cortical and subcortical regions (Nokia & Penttonen, 2022), which falls beyond the scope of our model. Addressing catastrophic forgetting in

connectionist models through energetics is beyond the focus of this study. Nonetheless, this remains an intriguing direction for future research.

Comment 7
Response to Comment 26.
Given that this is a paper about the role of plasticity, this doesn't seem to be a good rebuttal. I think it would be of help to the reader to see the weights evolving in time, akin to the firing rates. Reviewer 1 made a related request in their Comment 8 regarding plotting theoretical predictions on figures 6c/7c/8c. This hearkens back to my Comment 31 regarding Fig 4 as a schematic.. should Fig 4 not be computable for stated parameters values? Then figs 6c/7c/8c should be able to have simulations and theory agree.

Answer
We appreciate the suggestion. To address this, we have added a figure (Fig. S3 in the supplementary material) that illustrates the evolution of the average excitatory-excitatory synaptic weight values throughout the simulation. Additionally, we have included references to this figure in the main text to guide readers interested in the temporal dynamics of synaptic weights.

Comment 8
Response to Comment 27.
re: Heaviside. Sorry, my confusion was unrelated; I had made a mistake. But I agree, it's ok.

Answer
Thank you for the clarification.

Comment 9
Response to Comment 28.
re: solution locality (i.e. A \to 0 limit). I disagree; and I think a brief statement of this would make sense e.g. around Line 515 where you mention this limit, to ensure the reader knows that you're excluding it.

Answer
Thanks for the suggestion. We have added a brief clarification (Line 522) explicitly stating that we do not consider the A \to 0 limit in our analysis, ensuring that the reader is aware of this exclusion.

Comment 10
Response to Comment 37.
Yes, sorry, another mistake on my end; it's fine as-is.

Answer
No issue at all. We sincerely appreciate your careful review.

Reviewer #3: The paper can be accepted if the two other reviewers agree.

Answer
Thank you for your assessment. We appreciate your time and consideration.
